# Effect of birth type and sex on growth performance, wither height, humerus‐radius bone dimensions, humerus–ulna growth plate width and selected hormone profile in growing Gurcu goat kids

**DOI:** 10.1002/vms3.70013

**Published:** 2024-09-10

**Authors:** Buket Boğa Kuru, Enes Akyüz, Uğur Aydın, Mushap Kuru, Fikret Bektaşoğlu, Mert Sezer, Uğur Yıldız, Turgut Kırmızıbayrak

**Affiliations:** ^1^ Dep artment of Animal Breeding and Husbandry Faculty of Veterinary Medicine, Kafkas University Kars Turkey; ^2^ Department of Internal Medicine Faculty of Veterinary Medicine, Kafkas University Kars Turkey; ^3^ Department of Surgery Faculty of Medicine, Kafkas University Kars Turkey; ^4^ Department of Obstetrics and Gynecology Faculty of Veterinary Medicine, Kafkas University Kars Turkey

**Keywords:** body weight, epiphyseal plate, Gurcu goat, humerus, IGF‐I, thyroid hormones

## Abstract

**Objectives:**

In this study, the effects of sex and birth type on growth performance, withers height (WH), radiographic measurements and selected hormone profiles in Gurcu goat kids were investigated.

**Methods:**

Twenty kids (single female = 5, single male = 5, twin female = 5, twin male = 5) were included in the study. Body weight (BW), WH, radiographic measurements (humerus length [HL], radius length [RL], proximal humerus epiphyseal plate width [HEP] and distal ulna epiphyseal plate width [UEP]) and biochemical analysis (for serum calcitonin, free triiodothyronine [FT3], free thyroxine [FT4], growth hormone [GH] and insulin‐like growth factor‐I [IGF‐I]) were performed at 1, 3, 5, 7, 9 and 12 months of age.

**Results:**

BW was significantly higher in males starting from the seventh month compared to females (*p* < 0.05). HL was higher in males at seventh (*p* = 0.009) and ninth (*p* = 0.033) months, whereas RL was lower in twins at the third month (*p* = 0.021). UEP was wider in males at seventh (*p* = 0.008) and ninth (*p* = 0.036) months. Closure of HEP was observed in 65% of kids by the 12th month. Calcitonin was lower in twins at third (*p* = 0.045) and fifth (*p* = 0.006) months, with changes observed due to group and time effects (*p* < 0.05), whereas other hormones only changed with time (*p* < 0.05). Positive correlations were observed between BW, WH, HL, RL and IGF‐I. There was a negative correlation between BW, WH, HL, RL, IGF‐I and HEP, UEP, calcitonin, FT3, FT4, GH.

**Conclusion:**

Sex and birth type in Gurcu goat kids may have an impact on growth performance, radiographic measurements and certain hormonal profiles.

## Graphical abstract

This study found that sex and birth type can influence growth, bone development and hormone levels in Gurcu goat kids. Males grew faster and had wider growth plates than females. Twins had higher calcitonin levels at younger ages. Growth hormone and thyroid hormones changed with age, whereas IGF‐I levels were positively correlated with growth.

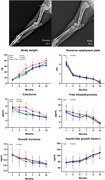

## 1 INTRODUCTION

Originating from the Caucasus, the Gurcu goat is a robust local breed that exhibits high tolerance to the challenging climate and geographical conditions of Eastern Anatolia (Kuru & Boğa Kuru, 2020). These goats are rarely affected by reproductive problems (Kuru et al., 2017) or metabolic diseases, presenting a potentially valuable option for regional farmers as an alternative to large‐scale cattle breeding. Unfortunately, their population growth has not reached the desired level (Kuru et al., 2024).

Goats excel in digesting cellulose‐based feeds, resisting diseases, navigating various terrains and displaying economic efficiency across diverse conditions (Gül et al., 2022; Henry et al., 2018). Sustainable animal production requires pinpointing factors impacting young animal growth. Identifying measurable environmental effects on productivity improves breeding animal selection by standardizing performance values (Gül et al., 2022). Various environmental factors play a significant role in kids growth, with birth type and sex standing out as particularly influential. Research has shown that singleton kids typically experience faster growth rates than twins. Additionally, male kids exhibit an innate growth capacity higher than that of females (Akbaş et al., 2013; Ceyhan et al., 2022; Gül et al., 2016; Keskin et al., 2017; Nuntapaitoon et al., 2021). The genes governing the sex and birth type of offspring are distinct from those regulating growth and development (Hanoglu Oral et al., 2023). Therefore, adjustments considering the growth and developmental traits of animals chosen for breeding could mitigate the impact of environmental factors, thereby augmenting the precision of selection (Gül et al., 2022; Shrestha & Fahmy, 2007).

Bones can be categorized into five types based on their shape: long, short, flat, irregular and sesamoid (Fails & Magee, 2018). Long bones, characterized by their length, have a cylindrical shaft (diaphysis) with capped ends (epiphyses). Examples include the humerus, femur, tibia and others in the limbs, providing support and movement (Fails & Magee, 2018; Liebich et al., 2020). Endochondral ossification, a vital process in bone development, involves five key steps: (1) formation of a bone collar, (2) development of cavities within the cartilage, (3) blood vessel invasion into the cartilage model, (4) lengthwise growth and (5) ossification of the growth plates (epiphyses) (Atabo et al., 2017; Setiawati & Rahardjo, 2019). The cartilage separating the primary and secondary ossification centres has several names: epiphyseal plate, growth plate, physis or epiphyseal cartilage (Anderson & Shapiro, 2010). Furthermore, the physiology and closure timing of the growth plate are also influenced by a multitude of factors (Ağirdil, 2020). For example, during puberty, skeletal growth decelerates or ceases altogether, ultimately leading to the closure of the growth plate (Ağirdil, 2020; Emons et al., 2011). Studies have indicated that the width and closure time of the epiphyseal plate in goats can vary depending on both age and breed (Alpdogan & Genccelep, 2012; Atabo et al., 2018; Karasu et al., 2018; Rahimzadeh, 2019). Studies have also been conducted on topics such as comparing the closure of the growth plate with age in Korean native goats (Choi et al., 2006) and the significance of radiographic images of the humerus for age‐determination in goats (Youssef et al., 2016). Additionally, the longitudinal and transverse growth of the radius–ulna bone in Red Sokoto goats has been monitored using radiographic morphometric measurements (Atabo et al., 2018, 2017). The data obtained from radiographic measurements of bones can be utilized to construct a guide for age estimation. This approach has the potential to address the prevalent issue of irregular birth record keeping among farmers. Furthermore, understanding the anatomy of longitudinal and transverse bone growth can be an asset to veterinarians in their diagnostic, prognostic and surgical procedures (Atabo et al., 2017; Choi et al., 2006).

The thyroid hormones (TH), triiodothyronine (T3) and thyroxine (T4) play a crucial role in various vital physiological functions in animals (Abdoun et al., 2018; Todini et al., 2022). These hormones are essential for maintaining the health and productivity of animals, influencing development, growth, metabolic activity, productive performance and regulation of parameters such as heart rate and body temperature (Gupta & Mondal, 2021; Todini et al., 2006). Current research clearly demonstrates the significant role of T3 and T4 hormones in the growth and development of domestic animals (Abdoun et al., 2018; Khaleghnia et al., 2021; Todini et al., 2022). In particular, T3 and T4 in small ruminants play a vital role in the productive performance of farm animals, including milk production, growth and fibre quality (Ismail & Al‐Hamdi, 2022; Joy et al., 2020). TH blood concentrations may serve as a potential indicator of an animal's metabolic and nutritional status (Todini et al., 2022). In studies investigating the effect of sex on TH activity in lambs and kids, conflicting results have been obtained (Ismail & Al‐Hamdi, 2022; Meena et al., 2024; Valavi et al., 2022).

Produced by the C cells of the thyroid gland, calcitonin, a peptide hormone, acts in opposition to parathyroid hormone (PTH), lowering blood calcium concentrations (Xie et al., 2020). Its most prominent effect is to decrease serum calcium and phosphorus concentrations and reduce calcium mobilization from the bone (Naot et al., 2020). Additionally, calcitonin may not vary according to sex, breed or birth season. In newborns, blood calcitonin concentrations surprisingly increase after birth, peaking within 24–48 h. Subsequently, it declines to childhood levels within a month. This early rise is believed to counteract the bone‐resorbing effect of PTH (Namgung & Tsang, 2017). Lambs, however, exhibit a different pattern, with calcitonin concentration remaining high for up to 6 weeks after birth (Garel et al., 1974).

Growth hormone (GH), a significant peptide hormone, supports growth, cell proliferation and renewal (Kumar et al., 2024). The balance between energy production and utilization in goats determines plasma GH concentration (Hirayama & Katoh, 2004). Synthesized in the anterior pituitary gland and released into the bloodstream, GH has direct or indirect effects on anabolic processes such as protein synthesis in many tissues. It regulates lipid metabolism, carbohydrate metabolism and body growth, playing a vital role in animal production, especially in livestock (Buranakarl et al., 2024). During post‐natal and puberty periods, GH and insulin‐like growth factor‐I (IGF‐I) play a critical role in determining longitudinal skeletal growth (Isaksson et al., 1987; van der Eerden et al., 2003). Deficiencies in GH can lead to serious growth‐related problems, such as stunted stature (Giustina et al., 2008). The activity of GH occurs through IGF‐I synthesized in the liver (Buranakarl et al., 2021). IGF‐I plays a crucial role in various physiological processes, encompassing reproduction, growth, lactation and overall organismal health (Al‐Samerria & Radovick, 2021; Pehlivan, 2019). Primarily synthesized in the liver, IGF‐I is also produced in an autocrine/paracrine manner by peripheral tissues such as the skin, ovary, placenta, breast and bone (Laron, 2001; Zhao et al., 2024). Implicated in the growth and functioning of virtually every organ in the body, IGF‐I predominantly stimulates post‐natal body growth (Al‐Samerria & Radovick, 2021; Rasouli et al., 2017).

In this study, the effects of sex and birth type on growth performance, withers height (WH), humerus and radius bone dimensions, humerus and ulna growth plate width, and selected hormone profile were investigated in growing Gurcu goat kids. Correlations between the obtained morphological and radiographic measurements and the selected hormone profile were examined in the kids until they reached one year of age. Studies evaluating these long‐term parameters together, revealing their changes and correlations, are quite limited, and it is known that no such study has been conducted on Gurcu goat kids previously.

## 2 MATERIALS AND METHODS

### 2.1 Location and climatic conditions

The study was conducted at the Kafkas University Faculty of Veterinary Medicine Education, Research and Application Farm. Kars experiences a continental climate within the Northeast Anatolia region. Within the study period, February was the coldest month, whereas June was the warmest. The highest and lowest average temperatures according to the months sampled in our study are provided in Table 1.

**TABLE 1:** The highest and lowest average temperatures during the study period.

Month	Average low (°C)	Average high (°C)
March	−7	4
May	4	16
July	9	25
September	5	22
November	−5	7
February	−13	−2

### 2.2 Animals

In this study, a total of 30 Gurcu goats aged 2–3 years, weighing between 38 and 42 kg, with a body condition score ranging from 2.5–3.5 (1 = thin, 5 = obese), which had previously given birth to at least one kid and successfully completed the post‐partum period, were selected. Oestrus synchronization was applied to these goats, and five bucks were used for mating during oestrus detection. Additionally, female (*n* = 5) and male (*n* = 5) singleton kids, as well as female (*n* = 5) and male (*n* = 5) twin kids born from these goats, were included in the study. Selected goats, bucks and kids received routine internal and external parasite treatments and vaccinations throughout the study period.

### 2.3 Oestrus synchronization protocol

Oestrus synchronization was performed to ensure that kidding occurred at similar times among the studied goats. This approach facilitated the inclusion of kids with similar birth times, thereby simplifying sampling and implementation procedures.

During the breeding season, a protocol utilizing sponges containing medroxyprogesterone acetate, equine chorionic gonadotropin (eCG) and d‐cloprostenol was employed to achieve oestrus synchronization. Seven days after the insertion of a progesterone‐impregnated sponge (60 mg, medroxyprogesterone acetate, Esponjavet, Hipra Animal Health) into the vagina, 400 IU eCG (i.m., Oviser, Hipra Animal Health) and 75 μg d‐cloprostenol (i.m., Gestavet Prost, Hipra Animal Health) were injected. The sponges were removed on the ninth day, and the goats were introduced to bucks.

Approximately 35 days after mating, pregnancy diagnosis was performed using transrectal ultrasonography (5–7.5 MHz, Draminski iScan, Draminski) to confirm embryo detection and identify pregnant goats (Kuru et al., 2018). A second pregnancy examination was conducted 55–60 days after mating, revealing a final total of 21 pregnant goats. From these pregnant goats, 11 single kids and 10 twin kids were born, but only 20 kids (5 single females, 5 single males, 5 twin females and 5 twin males) born around the same time were included in the study.

### 2.4 Nutrition of kids

No additional care or feeding regimen was introduced for the goats and kids during the study. The research instead adhered to the standard farm feeding program routinely implemented by the management.

The included kids under study remained with their dams for the initial 3 months. During this period, they were fed a mixed ration of 140 g of meadow hay and 261 g of concentrated feed per kid in addition to maternal milk. Between 3 and 12 months of age, the kids were granted access to pasture whenever weather conditions permitted. During daylight hours, the kids freely grazed, benefiting from the sunlight and returned to their shelter in the evening. When grazing was not possible during the day or when seasonal conditions were not suitable for grazing, the kids were offered a mixed ration. This ration consisted of 200 g of meadow hay and 530 g of concentrate feed per kid. Throughout the study period, water was provided ad libitum. The composition of the mixed ration and its nutritional content information, organized according to the sampled months, are outlined in Table 2.

**TABLE 2:** Proportions of ingredients in diets for different age groups.

Ingredient, g/kg DM	0–3 months	3–9 months
Meadow hay	140	200
Barley	50	100
Corn	30	200
Sunflower oil	1	50
Wheat bran	50	50
Molasses, beet	10	10
Cottonseed meal, 37%	50	30
Soybean meal, 44%	70	90
Dicalcium phosphate	1	‐
Limestone	1	13
Salt	1	1
Vitamin–mineral premix^a^	1	1

Calculated nutrient content, %DM		
Nutrient	0–3 months	3–9 months
ME, kcal/kg	2565	2825
Crude protein, %	19.80	17.60
Ca, %	0.99	0.89
P, %	0.45	0.40

Abbreviations: DM, dry matter, ME, metabolizable energy.

^a^ Contents of vitamin and mineral premix in 1 kg: 100 g of Na, 33 mg of I, 7 mg of Ca, 27 mg of Se, 3000 mg of Fe, 2660 mg of Mn, 167 mg of Cu, 10,000 mg of α‐tocopherol, 300,000 IU of cholecalciferol and 3000,000 IU of retinol.

### 2.5 Study groups

The study included data from 20 kids born at similar times following oestrus synchronization. Only clinically healthy kids who had ingested colostrum were selected. Blood samples were collected from kids at 1, 3, 5, 7, 9 and 12 months of age in each group.

Group 1 comprised five female kids born as singletons.

Group 2 included five male kids born as singletons.

Group 3 consisted of five female kids born as twins.

Group 4 contained five male kids born as twins.

### 2.6 Measurement of body weight and withers height

The body weight (BW) of the kid was measured by weighing it using a digital weighing scale (TEM Eko+ W1, Tüm Elektronik Mühendislik) at 1, 3, 5, 7, 9 and 12 months of age. The WH of the kid was measured while standing, and it was measured as the distance from the highest point of the withers to the base of the hooves using a measuring stick (Hauptner and Herberholz GmbH).

### 2.7 Radiographic measurements

**FIGURE 1:**
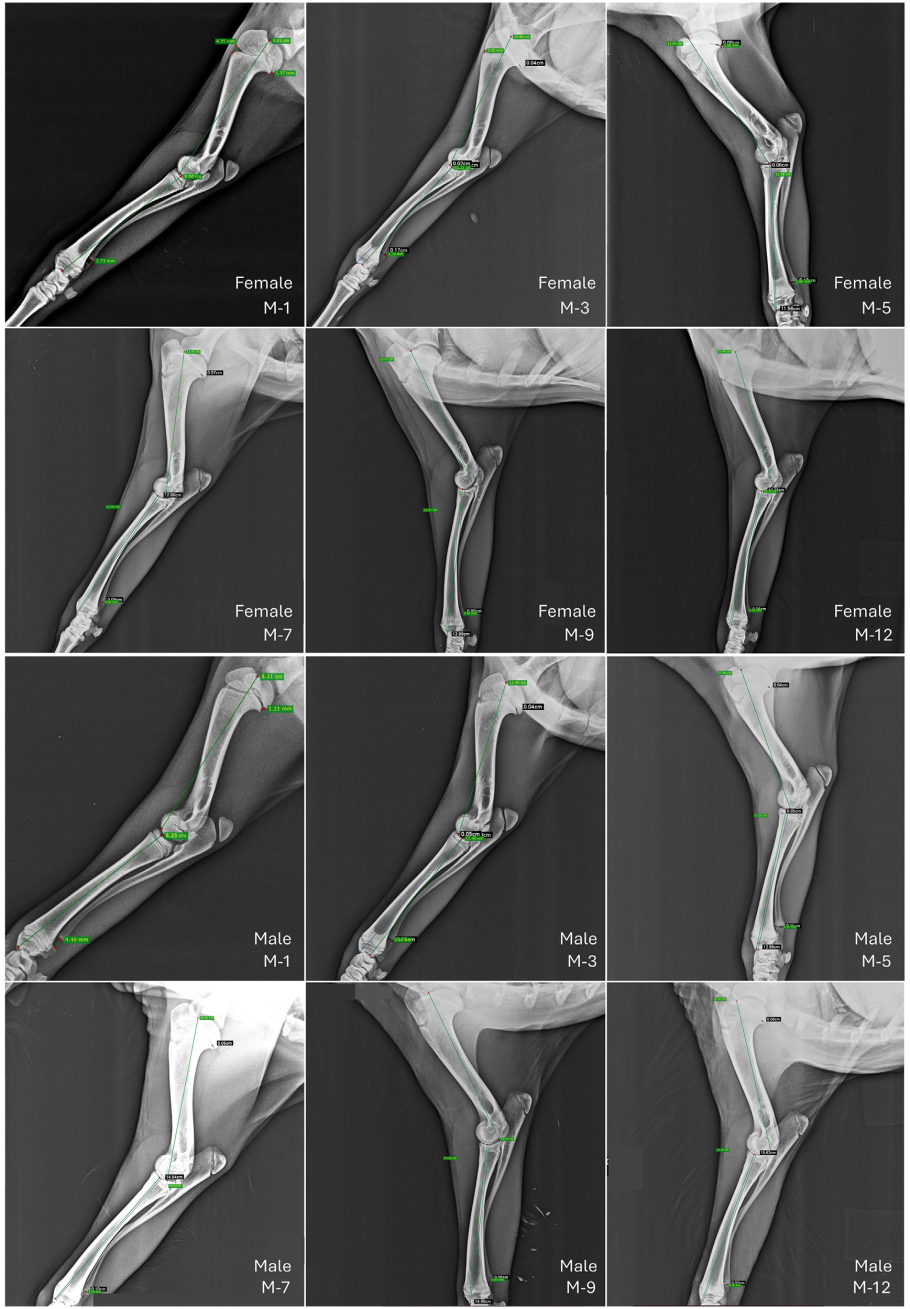
Radiographic measurements of humerus and radius length, and humerus and ulna epiphyseal plate width in growing female and male Gurcu goat kids by month of age. M, month.

Radiographic images were obtained using a 35 × 43 cm^2^ cassette. Kids were positioned in either right or left lateral recumbency. The images encompassed the humerus, radius–ulna complex, proximal humeral growth plate and distal ulnar growth plate. Exposure settings were 50 kV and 2.5 mAs, utilizing a computed radiography device (Fujifilm FCR Prima T2 Veterinary Set, Medical Technology). Measurements were taken at 1, 3, 5, 7, 9 and 12 months of age without anaesthesia. After acquiring the radiographic images, the device's built‐in measurement program was used to determine the distance between the caput humeri and trochlea humeri for humerus length (HL), and the distance between the caput radii and facies articularis carpalis for radius length (RL). Additionally, the widths of the proximal humeral growth plate and distal ulnar growth plates were measured regularly up to 12 months of age, and their closure status was monitored (Figure 1).

### 2.8 Blood collection

All blood samples from kids were collected in the morning either before feeding or immediately prior to turning out onto pasture. Blood samples were collected from the jugular vein into vacutainer tubes (BD Vacutainer, Becton, Dickinson and Company) at 1, 3, 5, 7, 9 and 12 months of age. The collected blood was centrifuged at 1500 *g* for 10 min at 4°C (NF 400R, Nüve). The obtained serum samples were stored in microcentrifuge tubes at −18°C for biochemical analyses.

### 2.9 Biochemical analysis

Serum free T3 (FT3) and free T4 (FT4) concentrations were measured using a fully automated chemiluminescent immunoassay (CLIA) method on a Siemens ADVIA Centaur XP (Siemens Healthcare Diagnostics) analyser with a compatible commercial kit.

Serum calcitonin, GH and IGF‐I concentrations were measured using a fully automated CLIA method on a Siemens IMMULITE 2000 XPI (Siemens Healthcare Diagnostics S.L.) analyser with commercial kits.

### 2.10 Statistical analysis

A priori power analysis was conducted using G*Power software (Version 3.1.9.7, Franz Faul, Universitat Kiel, Germany) to determine the appropriate sample size. The analysis was based on a power of 0.95, a significance level of 0.05 and an effect size (d) of 2.39, as reported in the literature (Pehlivan, 2019).

Data normality within each group was assessed using the Shapiro–Wilk test. General Linear Model was then employed to analyse the effects of sex and birth type (single and twin) on growth performance in the kids, encompassing WH, radiographic measurements and biochemical parameters. For parameters measured up to 12 months of age, a two‐way analysis of variance was conducted in the groups (single female, single male, twin female and twin male) to identify group, time and group × time interactions. Tukey's multiple comparison test was used for pairwise comparisons of both the differences among different measurement times within a group and the differences between groups at the same time point. To evaluate potential differences in the closure of the humerus growth plates at 9 and 12 months of age, the chi‐square test was utilized, taking into account the influences of group, sex and birth type. Pearson correlation coefficients were also determined among variables measured during the growth period in the kids. Data are presented as mean ± standard deviation. Statistical analyses were performed using GraphPad Prism (Version 9.5.1, GraphPad Software Inc., USA) and SPSS (Version 26.0, SPSS Inc./IBM Group, USA) software. A significance level of *p* < 0.05 was set for group comparisons.

## 3 RESULTS

### 3.1 Growth performance and wither height changes

Sex had a statistically significant effect on the growth performance of Gurcu goat kids at 7 months of age and beyond (*p* < 0.05). Birth type had significant effects on growth performance at 3 (*p* = 0.013) and 5 (*p* = 0.004) months of age (Table 3). Additionally, the effects of group (*p* = 0.01), time (*p* < 0.001) and group × time interaction (*p* < 0.001) were statistically significant for growth performance (Figure 1). Pairwise comparisons of intergroup statistical differences at 5, 7 and 12 months of age, as well as intragroup statistical differences over time for growth performance, are presented in Table S1.

**TABLE 3:** The effect of sex and birth type on growth performance and wither height in Gurcu goat kids.

Live weight and wither height		Age (months)					
		1	3	5	7	9	12
Growth performance (g)	Female (*n* = 10)	6.06 ± 1.18	12.23 ± 2.89	18.53 ± 3.57	25.86 ± 3.08^a^	30.03 ± 3.09^a^	32.04 ± 2.88^a^
	Male (*n* = 10)	6.81 ± 1.22	13.21 ± 4.40	22.12 ± 6.31	29.76 ± 4.28^b^	33.23 ± 3.78^b^	37.99 ± 4.55^b^
	Single (*n* = 10)	6.37 ± 1.43	14.71 ± 4.02^A^	23.35 ± 5.87^A^	28.96 ± 4.76	32.74 ± 3.85	36.0 ± 5.16
	Twin (*n* = 10)	6.5 ± 1.08	10.73 ± 1.83^B^	17.3 ± 2.24^B^	26.66 ± 3.27	30.52 ± 3.27	34.03 ± 4.45
	Total (*n* = 20)	6.43 ± 1.23	12.72 ± 3.66	20.32 ± 5.32	27.81 ± 4.14	31.63 ± 3.74	35.02 ± 4.80
	Sex effect	0.191	0.505	0.064	0.024	0.034	0.002
	Birth type effect	0.817	0.013	0.004	0.162	0.128	0.243
	Sex × birth type	0.274	0.391	0.188	0.162	0.067	0.151
Wither height (cm)	Female (*n* = 10)	36.75 ± 2.20	45.50 ± 2.37^a^	49.85 ± 1.55^a^	53.55 ± 1.76^a^	56.90 ± 2.17^a^	59.25 ± 2.73^a^
	Male (*n* = 10)	38.15 ± 2.01	48.40 ± 4.05^b^	54.05 ± 4.74^b^	58.05 ± 4.31^b^	62.05 ± 5.02^b^	64.45 ± 4.83^b^
	Single (*n* = 10)	37.40 ± 2.72	49.05 ± 3.61^A^	53.75 ± 4.58^A^	56.40 ± 5.18	60.25 ± 6.30	62.15 ± 6.04
	Twin (*n* = 10)	37.50 ± 1.62	44.85 ± 1.97^B^	50.15 ± 2.54^B^	55.20 ± 2.30	58.70 ± 1.89	61.55 ± 3.03
	Total (*n* = 20)	37.45 ± 2.18	46.95 ± 3.56	51.95 ± 4.05	55.80 ± 3.95	59.48 ± 4.60	61.85 ± 4.66
	Sex effect	0.051	0.015	0.003	0.005	0.006	0.011
	Birth type effect	0.882	0.001	0.009	0.404	0.357	0.744
	Sex × birth type	0.055	0.078	0.029	0.093	0.090	0.361

*Note*: Different letters (a and b) in the same column indicate statistical differences between females and males. Different letters (A and B) in the same column indicate statistical differences between single and twin kids.

**FIGURE 2:**
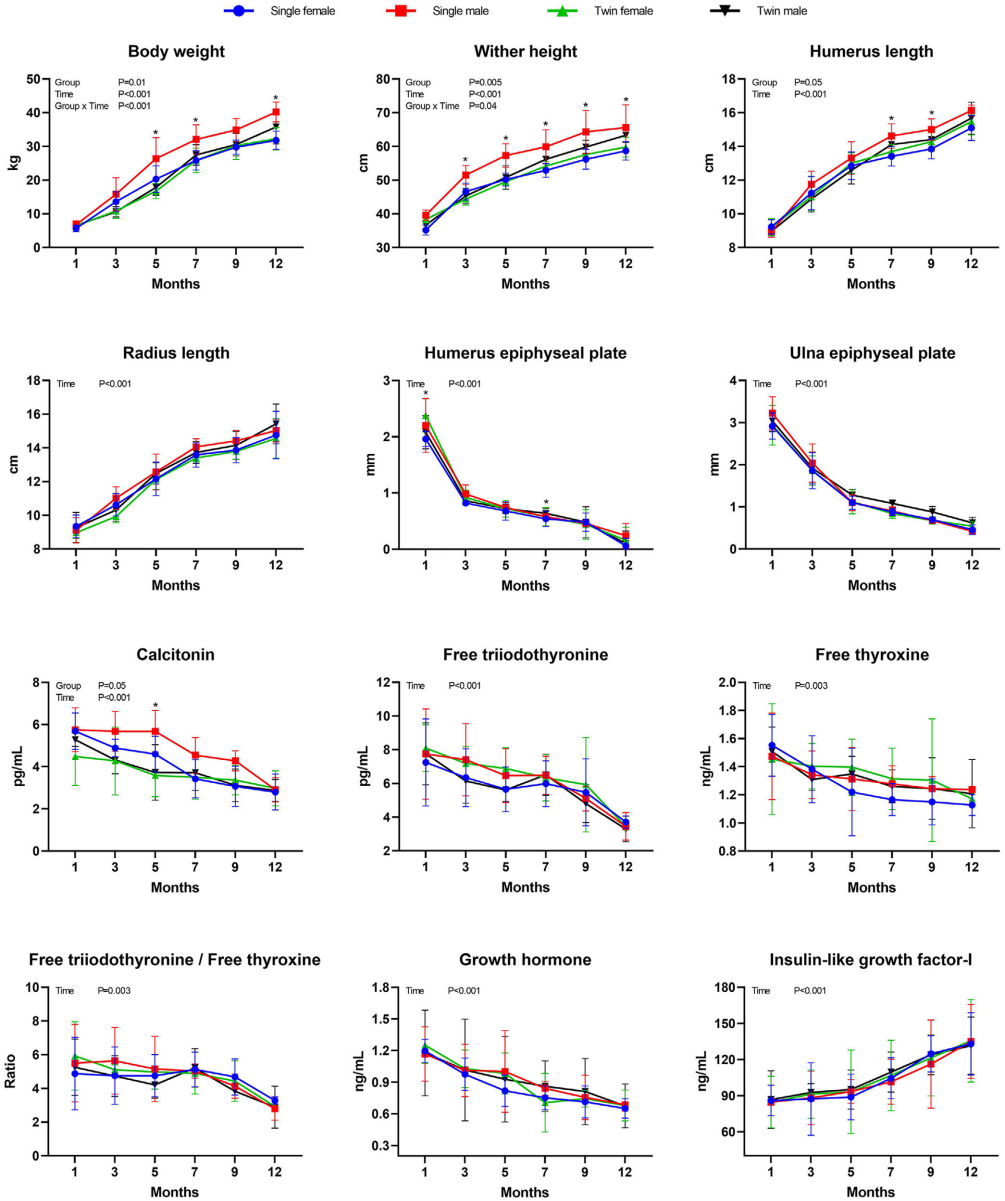
Growth performance, withers height, radiographic measurements and hormonal changes according to birth type and sex (single female, *n* = 5; single male, *n* = 5; twin female, *n* = 5; twin male, *n* = 5) over months. *: Indicates a statistically significant difference between groups in the month of measurement.

The height at the withers was statistically influenced by sex at 3 months of age and beyond (*p* < 0.05). Birth type also had a statistically significant effect at 3 (*p* = 0.001) and 5 (*p* = 0.009) months of age. Furthermore, the interaction between sex and birth type was statistically significant at 5 months of age for WH (*p* = 0.029) (Table 3). Additionally, group effect (*p* = 0.005), time effect (*p* < 0.001) and group × time interactions were statistically significant for WH (Figure 2). In particular, significant differences among groups emerged at 3 months of age and beyond (Figure 2 and Table S2), and intragroup time‐dependent statistical differences also occurred (Table S2).

### 3.2 Radiographic measurements

In Gurcu goat kids, the sex effect on HL was significant at 7 (*p* = 0.009) and 9 months (*p* = 0.033). However, for RL, birth type had a statistically significant effect at 3 months (*p* = 0.021). Birth type had no statistically significant effect on HL, and sex had no statistically significant effect on RL (Table 4). Moreover, the group effect (*p* = 0.05) and time effect (*p* < 0.001) were significant for HL. For RL, only the time effect (*p* < 0.001) was significant (Figure 2). HL was statistically different between single female kids and single male kids at 7 (*p* = 0.03) and 9 (*p* = 0.05) months of age (Table S3). Pairwise comparisons of both humerus and RL within groups over time are provided in Tables S3 and S4, respectively.

**TABLE 4:** The effect of sex and birth type on humerus‐radius length and epiphyseal plate closure in Gurcu goat kids.

Bone and epiphyseal plate		Age (months)					
		1	3	5	7	9	12
Humerus length (cm)	Female (*n* = 10)	9.22 ± 0.44	11.12 ± 0.87	12.93 ± 0.69	13.56 ± 0.49^a^	14.07 ± 0.57^a^	15.28 ± 0.75
	Male (*n* = 10)	8.95 ± 0.28	11.32 ± 0.86	12.95 ± 0.92	14.37 ± 0.72^b^	14.71 ± 0.70^b^	15.90 ± 0.71
	Single (*n* = 10)	9.08 ± 0.39	11.49 ± 0.88	13.08 ± 0.88	14.02 ± 0.88	14.43 ± 0.83	15.61 ± 0.76
	Twin (*n* = 10)	9.09 ± 0.40	10.95 ± 0.76	12.79 ± 0.72	13.91 ± 0.58	14.34 ± 0.58	15.57 ± 0.83
	Total (*n* = 20)	9.08 ± 0.39	11.22 ± 0.85	12.94 ± 0.79	13.96 ± 0.73	14.39 ± 0.70	15.59 ± 0.78
	Sex effect	0.143	0.617	0.957	0.009	0.033	0.077
	Birth type effect	0.982	0.170	0.436	0.692	0.749	0.912
	Sex × birth type	0.911	0.397	0.241	0.168	0.079	0.227
Radius length (cm)	Female (*n* = 10)	9.16 ± 0.54	10.28 ± 0.63	12.15 ± 0.77	13.49 ± 0.52	13.83 ± 0.59	14.67 ± 1.22
	Male (*n* = 10)	9.19 ± 0.78	10.66 ± 0.74	12.53 ± 0.83	13.90 ± 0.57	14.29 ± 0.70	15.23 ± 0.93
	Single (*n* = 10)	9.23 ± 0.68	10.82 ± 0.66^A^	12.37 ± 0.99	13.82 ± 0.63	14.15 ± 0.70	14.90 ± 1.05
	Twin (*n* = 10)	9.12 ± 0.66	10.12 ± 0.56^B^	12.31 ± 0.61	13.57 ± 0.50	13.98 ± 0.67	15.00 ± 1.20
	Total (*n* = 20)	9.18 ± 0.66	10.47 ± 0.70	12.34 ± 0.80	13.70 ± 0.57	14.06 ± 0.67	14.95 ± 1.10
	Sex effect	0.909	0.176	0.323	0.127	0.153	0.286
	Birth type effect	0.728	0.021	0.872	0.325	0.581	0.850
	Sex × birth type	0.398	0.989	0.950	0.771	0.744	0.564
Humerus epiphyseal plate (mm)	Female (*n* = 10)	2.17 ± 0.31	0.87 ± 0.11	0.70 ± 0.15	0.56 ± 0.14	0.46 ± 0.21	0.11 ± 0.19
	Male (*n* = 10)	2.14 ± 0.38	0.92 ± 0.13	0.73 ± 0.08	0.61 ± 0.07	0.47 ± 0.19	0.17 ± 0.22
	Single (*n* = 10)	2.08 ± 0.36	0.90 ± 0.14	0.71 ± 0.13	0.56 ± 0.10	0.47 ± 0.12	0.15 ± 0.20
	Twin (*n* = 10)	2.23 ± 0.32	0.89 ± 0.10	0.72 ± 0.11	0.61 ± 0.13	0.46 ± 0.25	0.13 ± 0.22
	Total (*n* = 20)	2.16 ± 0.34	0.90 ± 0.12	0.72 ± 0.12	0.59 ± 0.11	0.47 ± 0.19	0.14 ± 0.20
	Sex effect	0.839	0.327	0.603	0.355	0.916	0.523
	Birth type effect	0.317	0.842	0.862	0.355	0.916	0.831
	Sex × Birth type	0.082	0.051	0.603	0.851	0.753	0.210
Ulna epiphyseal plate (mm)	Female (*n* = 10)	2.93 ± 0.38	1.86 ± 0.37	1.11 ± 0.22	0.86 ± 0.10^a^	0.69 ± 0.07^a^	0.50 ± 0.12
	Male (*n* = 10)	3.13 ± 0.33	1.98 ± 0.40	1.19 ± 0.18	0.99 ± 0.13^b^	0.78 ± 0.14^b^	0.52 ± 0.14
	Single (*n* = 10)	3.07 ± 0.37	1.95 ± 0.43	1.10 ± 0.16	0.89 ± 0.09	0.69 ± 0.06^A^	0.44 ± 0.08^A^
	Twin (*n* = 10)	2.99 ± 0.36	1.89 ± 0.34	1.20 ± 0.23	0.96 ± 0.16	0.78 ± 0.15^B^	0.58 ± 0.12^B^
	Total (*n* = 20)	3.03 ± 0.36	1.92 ± 0.38	1.15 ± 0.20	0.93 ± 0.13	0.74 ± 0.12	0.51 ± 0.13
	Sex effect	0.241	0.517	0.383	0.008	0.036	0.679
	Birth type effect	0.633	0.745	0.279	0.123	0.036	0.009
	Sex × birth type	0.551	0.745	0.383	0.021	0.013	0.224

*Note*: Different letters (a and b) in the same column indicate statistical differences between females and males. Different letters (A and B) in the same column indicate statistical differences between single and twin kids.

Sex and birth type had no significant effect on humerus epiphyseal plate width (HEP). However, sex influenced ulna epiphyseal plate width (UEP) at 7 (*p* = 0.008) and 9 months (*p* = 0.036), whereas birth type was statistically significant at 9 (*p* = 0.036) and 12 months (*p* = 0.009) (Table 4). Furthermore, the interaction between sex and birth type was significant for UEP at 7 (*p* = 0.021) and 9 months (*p* = 0.013) (Table 4). Although group effect was not significant (*p* > 0.05) for either humerus or UEP, the time effect was statistically significant (*p* < 0.001) (Figure 2). However, in pairwise comparisons, HEP was statistically different between single female and twin female kids in the first month (*p* = 0.006) (Table S5). Pairwise comparison of the temporal changes in humerus and UEP within groups is provided in Tables S5 and S6, respectively.

In all measurement processes, humerus epiphyseal plates remained mostly open for up to 9 months, with closure occurring in only two kids at that time point. By the 12th month, a total of 13 kids (65%) had closed humerus epiphyseal plates (Table 5). However, ulna epiphyseal plates did not close in any of the kids.

**TABLE 5:** Closure rates of humerus growth plate in Gurcu goat kids at 9 and 12 months of age according to groups.

Groups	Month 9	Month 12
	% (*n*/total *n*)	% (*n*/total *n*)
Single female	0 (0/5)	80 (4/5)
Single male	0 (0/5)	40 (2/5)
Twin female	20 (1/5)	60 (3/5)
Twin male	20 (1/5)	80 (4/5)
Female	10 (1/10)	70 (7/10)
Male	10 (1/10)	60 (6/10)
Single	0 (0/10)	60 (6/10)
Twin	20 (2/10)	70 (7/10)
Total	10 (2/20)	65 (13/20)

### 3.3 Biochemical changes

In Gurcu goat kids, birth type significantly affected calcitonin concentration at 3 (*p* = 0.045) and 5 months (*p* = 0.006), and the interaction between sex and birth type was significant at 9 months (*p* = 0.015) for calcitonin concentration only. The effect of sex and birth type on other hormones was nonsignificant (Table 6). Furthermore, although group (*p* = 0.05) and time (*p* < 0.001) effects were significant for calcitonin concentration, only time was statistically significant for other hormones (Figure 2). At 5 months, calcitonin concentration was statistically different in single males compared to twin males and females (Table S7). Pairwise comparisons of the variation in hormone concentration according to the sampling time are provided in Tables S7–S11.

**TABLE 6:** The effect of sex and birth type on some hormone concentrations in Gurcu goat kids.

Hormones		Age (months)					
		1	3	5	7	9	12
Calcitonin (pg/mL)	Female (*n* = 10)	5.08 ± 1.25	4.58 ± 1.15	4.09 ± 1.02	3.46 ± 0.92	3.22 ± 0.80	2.89 ± 0.80
	Male (*n* = 10)	5.52 ± 0.77	5.00 ± 1.05	4.70 ± 1.50	4.12 ± 0.90	3.60 ± 0.84	2.89 ± 0.52
	Single (*n* = 10)	5.72 ± 0.90	5.28 ± 0.81^A^	5.14 ± 1.03^A^	3.98 ± 1.01	3.67 ± 0.95	2.85 ± 0.69
	Twin (*n* = 10)	4.88 ± 1.03	4.30 ± 1.16^B^	3.65 ± 1.10^B^	3.60 ± 0.89	3.14 ± 0.61	2.92 ± 0.65
	Total (*n* = 20)	5.30 ± 1.03	4.79 ± 1.10	4.40 ± 1.29	3.79 ± 0.95	3.41 ± 0.82	2.89 ± 0.65
	Sex effect	0.335	0.367	0.210	0.122	0.229	1.000
	Birth type effect	0.073	0.045	0.006	0.361	0.098	0.828
	Sex × birth type	0.416	0.418	0.333	0.283	0.015	0.742
Free triiodothyronine (pg/mL)	Female (*n* = 10)	7.67 ± 2.01	6.77 ± 1.39	6.27 ± 1.37	6.16 ± 1.30	5.70 ± 2.30	3.57 ± 0.31
	Male (*n* = 10)	7.75 ± 2.16	6.78 ± 1.80	6.04 ± 1.24	6.50 ± 1.10	4.96 ± 0.91	3.37 ± 0.75
	Single (*n* = 10)	7.50 ± 2.49	6.88 ± 1.91	6.06 ± 1.45	6.23 ± 1.20	5.29 ± 1.43	3.58 ± 0.61
	Twin (*n* = 10)	7.93 ± 1.54	6.67 ± 1.23	6.25 ± 1.15	6.43 ± 1.23	5.37 ± 2.10	3.37 ± 0.53
	Total (*n* = 20)	7.71 ± 2.03	6.77 ± 1.57	6.15 ± 1.28	6.33 ± 1.19	5.33 ± 1.75	3.47 ± 0.57
	Sex effect	0.935	0.988	0.688	0.562	0.383	0.464
	Birth type effect	0.665	0.776	0.738	0.739	0.929	0.425
	Sex × birth type	0.677	0.162	0.081	0.783	0.654	0.832
Free thyroxine (ng/mL)	Female (*n* = 10)	1.50 ± 0.31	1.39 ± 0.19	1.31 ± 0.26	1.24 ± 0.18	1.23 ± 0.32	1.15 ± 0.06
	Male (*n* = 10)	1.49 ± 0.24	1.33 ± 0.12	1.33 ± 0.17	1.27 ± 0.12	1.24 ± 0.16	1.22 ± 0.16
	Single (*n* = 10)	1.51 ± 0.26	1.36 ± 0.20	1.27 ± 0.26	1.22 ± 0.13	1.20 ± 0.13	1.18 ± 0.08
	Twin (*n* = 10)	1.48 ± 0.29	1.36 ± 0.13	1.37 ± 0.16	1.29 ± 0.17	1.27 ± 0.33	1.19 ± 0.17
	Total (*n* = 20)	1.50 ± 0.27	1.36 ± 0.16	1.32 ± 0.22	1.25 ± 0.15	1.24 ± 0.25	1.19 ± 0.13
	Sex effect	0.927	0.388	0.829	0.671	0.883	0.225
	Birth type effect	0.806	0.939	0.307	0.347	0.516	0.905
	Sex × birth type	0.614	0.720	0.496	0.234	0.521	0.531
Free triiodothyronine/free thyroxine	Female (*n* = 10)	5.40 ± 2.05	4.94 ± 1.17	4.87 ± 1.08	5.02 ± 1.08	4.56 ± 1.08	3.11 ± 0.25
	Male (*n* = 10)	5.38 ± 1.90	5.19 ± 1.62	4.68 ± 1.47	5.13 ± 0.82	3.99 ± 0.58	2.84 ± 0.95
	Single (*n* = 10)	5.19 ± 2.12	5.20 ± 1.80	4.96 ± 1.55	5.08 ± 0.74	4.41 ± 0.91	3.04 ± 0.54
	Twin (*n* = 10)	5.59 ± 1.78	4.93 ± 0.85	4.60 ± 0.95	5.06 ± 1.13	4.14 ± 0.91	2.91 ± 0.83
	Total (*n* = 20)	5.39 ± 1.92	5.06 ± 1.38	4.78 ± 1.26	5.07 ± 0.93	4.27 ± 0.89	2.98 ± 0.69
	Sex effect	0.976	0.702	0.754	0.812	0.176	0.422
	Birth type effect	0.667	0.679	0.552	0.970	0.512	0.689
	Sex × birth type	0.488	0.343	0.338	0.659	0.950	0.490
Growth hormone (ng/mL)	Female (*n* = 10)	1.22 ± 0.14	1.00 ± 0.16	0.90 ± 0.18	0.73 ± 0.20	0.73 ± 0.12	0.67 ± 0.12
	Male (*n* = 10)	1.17 ± 0.32	1.01 ± 0.36	0.97 ± 0.38	0.85 ± 0.17	0.78 ± 0.25	0.68 ± 0.14
	Single (*n* = 10)	1.18 ± 0.19	0.99 ± 0.20	0.91 ± 0.29	0.80 ± 0.10	0.74 ± 0.17	0.67 ± 0.07
	Twin (*n* = 10)	1.22 ± 0.30	1.02 ± 0.34	0.96 ± 0.30	0.78 ± 0.26	0.78 ± 0.22	0.68 ± 0.17
	Total (*n* = 20)	1.20 ± 0.24	1.01 ± 0.27	0.93 ± 0.29	0.79 ± 0.19	0.76 ± 0.19	0.67 ± 0.13
	Sex effect	0.668	0.929	0.651	0.177	0.561	0.811
	Birth type effect	0.781	0.835	0.746	0.883	0.649	0.886
	Sex × birth type	0.846	0.858	0.396	0.710	0.890	0.762
Insulin‐like growth factor‐I (ng/mL)	Female (*n* = 10)	85.30 ± 16.59	89.24 ± 24.25	91.07 ± 26.45	105.66 ± 22.53	123.04 ± 23.31	134.30 ± 28.70
	Male (*n* = 10)	85.72 ± 15.98	90.42 ± 15.73	94.32 ± 12.62	105.57 ± 17.17	120.06 ± 27.07	133.29 ± 25.90
	Single (*n* = 10)	85.36 ± 8.47	87.71 ± 24.93	91.20 ± 14.43	103.01 ± 16.89	120.48 ± 26.80	134.00 ± 26.75
	Twin (*n* = 10)	85.66 ± 21.42	91.95 ± 14.32	94.19 ± 25.53	108.22 ± 22.40	122.62 ± 23.67	133.59 ± 27.91
	Total (*n* = 20)	85.51 ± 15.86	89.83 ± 19.90	92.70 ± 20.24	105.62 ± 19.49	121.55 ± 24.63	133.80 ± 26.61
	Sex effect	0.957	0.904	0.744	0.992	0.805	0.939
	Birth type effect	0.969	0.666	0.764	0.586	0.859	0.975
	Sex × birth type	0.836	0.977	0.889	0.762	0.645	0.819

*Note*: Different letters (A and B) in the same column indicate statistical differences between single and twin kids.

### 3.4 Pearson correlation coefficients between variables

The growth performance, WH, humerus and RL, and IGF‐I showed positive correlations among themselves. Similarly, positive correlations were observed between humerus and UEP, calcitonin, FT3, FT4, FT3/FT4 and GH. However, negative correlations were found between growth performance, WH, humerus and RL, and IGF‐I with humerus and UEP, calcitonin, FT3, FT4 and GH. The Pearson correlation coefficients and significance levels between variables are presented in Table 7.

**TABLE 7:** Pearson correlation coefficients between variables measured during the growth period in Gurcu goat kids (*n* = 120).

Parameters	Wither height	Humerus length	Radius length	Humerus EP	Ulna EP	Calcitonin	FT3	FT4	FT3/FT4	GH	IGF‐I
Growth performance	0.942^***^	0.914^***^	0.907^***^	−0.795^***^	−0.855^***^	−0.552^***^	−0.548^***^	−0.402^***^	−0.390^***^	−0.574^***^	0.588^***^
Wither height		0.917^***^	0.894^***^	−0.821^***^	−0.850^***^	−0.508^***^	−0.544^***^	−0.397^***^	−0.391^***^	−0.572^***^	0.501^***^
Humerus length			0.946^***^	−0.849^***^	−0.895^***^	−0.592^***^	−0.563^***^	−0.374^***^	−0.425^***^	−0.578^***^	0.526^***^
Radius length				−0.807^***^	−0.862^***^	−0.587^***^	−0.560^***^	−0.379^***^	−0.422^***^	−0.604^***^	0.562^***^
Humerus EP					0.902^***^	0.536^***^	0.584^***^	0.425^***^	0.425^***^	0.587^***^	−0.456^***^
Ulna EP						0.599^***^	0.513^***^	0.448^***^	0.334^***^	0.591^***^	−0.507^***^
Calcitonin							0.472^***^	0.323^***^	0.348^***^	0.307^***^	−0.455^***^
FT3								0.323^***^	0.878^***^	0.327^***^	−0.424^***^
FT4									−0.150	0.256^**^	−0.189^*^
FT3/FT4										0.244^**^	−0.361^***^
GH											−0.422^***^

Abbreviations: EP, epiphyseal plate; FT3, free triiodothyronine; FT4, free thyroxine; GH, growth hormone; IGF‐I, insulin‐like growth factor‐I.

* Correlation is significant at the 0.05 level (2‐tailed).

** Correlation is significant at the 0.01 level (2‐tailed).

*** Correlation is significant at the 0.001 level (2‐tailed).

## 4 DISCUSSION

The Gurcu goat is one of the local goat breeds of northeastern Türkiye. Previous studies have focused on the metabolic profile (Kuru et al., 2020, 2024; Ölmez et al., 2020), spermatological characteristics (Kulaksız et al., 2020, 2019) and some clinical, haematological and biochemical parameters (Akyüz et al., 2020) of these goats. However, to our knowledge, no study has yet investigated the growth performance, humerus‐RL, humerus–ulna growth plate width and growth‐related hormones (calcitonin, FT3, FT4, GH and IGF‐I) throughout the growth period in these goats. Additionally, our extensive literature review did not reveal any studies that examine the relationships between growth parameters, radiographic measurements and hormonal profiles in goats throughout their growth period.

In a study, male kids exhibited greater growth compared to female kids over a 5‐month period. Males also had significantly higher BWs at the end of the study (Pehlivan, 2019). A significant effect of both sex and birth type on growth performance has also been reported in goat kids (Gül et al., 2022). Additionally, sex and birth type may affect growth performance up to 6 months of age in goat kids, with these effects diminishing by the ninth month (Tozlu Çelik & Olfaz, 2018). Sex has a significant effect on growth performance in a study involving different goat breeds at the end of a 12‐week follow‐up period (Al‐Dawood et al., 2020). In this study, male Gurcu goat kids had significantly higher BWs and wither heights compared to females at 12 months of age. However, the same was not observed for birth type, with twins having slightly lower growth performance and wither heights compared to singletons, although not statistically significant. Nevertheless, a linear increase in BW and wither height over time was observed. In particular, there was a strong positive correlation between growth performance and wither height in Gurcu goat kids up to 12 months of age (*r* = 0.942, *p* < 0.001). The strong correlation between these two parameters suggests that wither height can be used as a reliable predictor of growth performance in Gurcu goat kids.

Bone size and epiphyseal plate width are considered reliable indicators for age estimation due to their steady growth patterns (Choi et al., 2006). Notably, both humerus and RL in goats increase with age (Atabo et al., 2017; Youssef et al., 2016). In this study, the time effect was found to be significant for both humerus and RL, showing an increase with age. Sex only affected HL in specific age ranges (e.g. 7–9 months). Additionally, a strong positive correlation was observed between measured bone lengths in Gurcu goat kids and WH and live weight. Investigations of epiphyseal plate width in goats consistently demonstrate a negative association with age (Atabo et al., 2018; Choi et al., 2006; Youssef et al., 2016). This study's measurements focused on relating epiphyseal plate width to age, indicating whether the plates were open or closed. In Gurcu goat kids, the width of the humeral epiphyseal plate continuously decreases with age.

In native Korean goats, the closure periods of the proximal humerus and distal ulna epiphyseal plates exceed 1 year (Choi et al., 2006). Marghoz goat kids showed sexual dimorphism in epiphyseal closure, with males closing later than females in both humerus (13.8 vs. 17.1 months) and ulna (15.5 vs. 17.3 months) (Rahimzadeh, 2019). The closure of the ulna distal epiphyseal plate varies in lambs, reported at 14–15 months (Gençcelep et al., 2002) and up to 34–35 months in kids (Genccelep et al., 2012), with males closing later (Popkin et al., 2012). Factors like breed, sex, nutrition and hormone issues can influence closure time (Gençcelep et al., 2002; Karasu et al., 2018). In Gurcu goat kids, consistent with other studies (Atabo et al., 2018; Choi et al., 2006; Genccelep et al., 2012; Rahimzadeh, 2019), the ulna epiphyseal plate remained open at 12 months. Additionally, in the first month, the birth type affected the HEP in female kids. Notably, 65% showed complete closure of the proximal humerus plate. A strong negative correlation existed between growth performance and both humerus (*r* = −0.795, *p* < 0.001) and ulna (*r* = −0.855, *p* < 0.001) epiphyseal plate widths. Therefore, both bone lengths and epiphyseal plate width in Gurcu goat kids could be presumed to be good indicators for age estimation, which our ongoing study continues to explore within age estimation modelling.

Calcitonin deficiency leads to a significant decrease in calcium and phosphate content in bone tissue, resulting in bone weakening and demineralization (Davey & Findlay, 2013; Felsenfeld & Levine, 2015; Kovacs & Kronenberg, 1997). Calcitonin increases during the first 48 h of life but may gradually approach adult levels within the first month after birth (Kovacs & Kronenberg, 1997; Ryan & Kovacs, 2020). Calcitonin concentration may be affected by sex and age. Although not all studies have found this, it is generally suggested that calcitonin concentration may decrease with age (Felsenfeld & Levine, 2015; Kiriakopoulos et al., 2022). In our study, serum calcitonin concentration did not significantly change during the first 5 months. In the kids (excluding twin female kids), the calcitonin concentration in the first month was higher than in the 12th month, and sex had no significant effect on calcitonin concentration. However, singleton male kids exhibited higher calcitonin concentration at 5 months compared to singleton and twin female kids. Additionally, calcitonin demonstrated strong correlations with growth‐related parameters, showing negative correlation with epiphyseal plate widths and positive correlation, indicating strong associations.

THs play a crucial role in growth and development by affecting cellular metabolism (Madan et al., 2019). Various factors, including season, nutrition, age, sex, climate, breed and physiological events, influence TH concentrations (Eshratkhah et al., 2010). In Saanen goat kids, T4 concentration declines significantly at 10 days of age and stabilizes until the third month, exceeding adult levels. T3 also shows a decreasing trend during the first 2 months of life. The T3/T4 ratio increases slightly from birth to day 10 and then decreases with age (Valavi et al., 2022). T3 concentration differs significantly between 1 and 12 months of age in Barbari goats, whereas both T3 and T4 exhibit similar patterns in Jamunapari goats (Bhooshan et al., 2010). T3 and T4 concentrations may gradually decrease in bucks up to 3 years of age (Nazifi et al., 2002). TH concentrations decrease with age in lambs, with no sex effect (Eshratkhah et al., 2010; Fırat et al., 2005; Ismail & Al‐Hamdi, 2022). Beetal goats exhibit high TH concentrations in early development, with no sex effect (Madan et al., 2019). Month, sex and live weight do not significantly impact T3 and T4 in Angora goats (Polat & Dellal, 2008). In the present study, no significant effects of sex or birth type on TH were revealed in Gurcu goat kids. However, a significant effect of age was observed, with TH concentration exhibiting a progressive decline as the kids matured. Notably, FT3 displayed a significant decrease between 1 and 12 months, whereas the reduction in T4 was less pronounced. Furthermore, TH demonstrated strong negative correlations with BW, wither height, bone lengths and IGF‐I, while exhibiting strong positive correlations with epiphyseal plate widths, calcitonin and GH. This study represents the first exploration of the combined effects of sex and birth type on TH in Gurcu goat kids. Previous research has documented the influence of various factors on TH concentration in different goat and sheep breeds (Eshratkhah et al., 2010; Madan et al., 2019; Valavi et al., 2022). Our findings align with reports indicating a decline in TH with increasing age in lambs (Eshratkhah et al., 2010; Fırat et al., 2005; Ismail & Al‐Hamdi, 2022) and kids (Bhooshan et al., 2010; Madan et al., 2019; Nazifi et al., 2002; Valavi et al., 2022). The observed negative correlations between TH and growth parameters (BW, wither height and bone lengths) and IGF‐I suggest a potential role for TH in regulating growth processes in Gurcu goat kids. Conversely, the positive correlations with epiphyseal plate widths, calcitonin and GH may indicate complex interactions between TH and these factors during skeletal development.

GH directly affects skeletal cells, with most of its effects mediated through IGF‐I, produced in peripheral tissues and present in the systemic circulation (Giustina et al., 2008; Isaksson et al., 1987). In our study, GH and IGF‐I were not influenced by sex and birth type in Gurcu goat kids, but they were affected by age. Although GH concentration showed a decreasing trend with age, IGF‐I concentration tended to increase. GH concentration was significant between single female kids at 1 and 12 months, and between twin female kids at 1 month and between 7 and 12 months. These findings suggest that GH secretion may vary differently in single and twin female Gurcu goat kids during the first year after birth. However, IGF‐I concentration was different between single males at 1 and 12 months. Positive correlations were observed between GH and epiphyseal plate width and TH, whereas IGF‐I showed positive correlations with growth performance, wither height and bone dimensions. Previous studies have shown that GH and IGF‐I exhibit varying concentrations depending on age and sex (Devrim et al., 2015; Hashizume et al., 1999; Kassim & Al‐Hellou, 2018; Pehlivan, 2019; Pragna et al., 2018). Breed, sex and time were effective on GH in Hair and Honamli goats (Devrim et al., 2015), whereas, in another study, breed was not effective on GH (Pragna et al., 2018). IGF‐I increased with age in goat kids. In both Hair and Honamli female kids, IGF‐I significantly increased after 4 months, whereas, in male kids, IGF‐I increased significantly after 4 months in Hair goat kids and after 8 months in Honamli goat kids (Devrim et al., 2015). In Shiba goat kids, GH concentrations were significantly higher in the first 1–2 weeks after birth compared to weeks 5–12. Similarly, IGF‐I was higher in the first 1–3 weeks after birth compared to weeks 6–12. Moreover, sex had no significant effect on both GH and IGF‐I (Hashizume et al., 1999). In sheep, GH concentration was higher in individuals under 1 year of age compared to those over 1 year of age (Kassim & Al‐Hellou, 2018). In White and Angora goats, IGF‐I tended to increase with age. IGF‐I differed between females and males in White goat kids, but not in Angora goat kids. Differences in IGF‐I between kids of the same sex from different breeds were also statistically significant. There were strong positive correlations between IGF‐I and live weight and wither height in male White goats and male–female Angora goats (Pehlivan, 2019). The findings from this study, combined with previous research results, indicate that GH and IGF‐I play significant roles in the growth and bone development of goat kids. However, the secretion of these hormones can vary depending on factors such as breed, sex, age and nutrition. We conducted measurements of these hormones in Gurcu goats for the first time, and a more comprehensive study could better elucidate possible relationships. This would help determine the effects on growth and bone development, as well as the roles of genetic and environmental factors.

There is evidence supporting a negative correlation between GH and IGF‐I in growing goats. GH and IGF‐I levels were lower in younger goats compared to older ones (Abdelsattar et al., 2022). Additionally, a decrease in plasma IGF‐I levels corresponded to a reduction in growth in growing goats (Sun et al., 2007). The relationship between GH and IGF‐I is crucial for regulating bone and muscle growth in livestock (Surya et al., 2021). Furthermore, GH plays a significant role in regulating the synthesis and secretion of IGF‐I, which is a principal mediator of the growth effects of GH (Switzer et al., 2009). The GH/IGF‐I axis is essential for skeletal growth, with both hormones having regulatory roles in this process (Isaksson et al., 1991). However, some studies suggest a negative correlation between baseline IGF‐I and the response to GH treatment in individuals with GH deficiency (Gibney & Johannsson, 2004; Lin et al., 2009; Sugimoto & Chihara, 1996). Additionally, insights into the dynamic interaction among GH, prolactin and IGF‐I during gestation, lactation and the neonatal period in goats could shed light on the negative correlation between GH and IGF‐I in growing goats (Hashizume et al., 1999). Although our study acknowledges the potential impact of sampling days on GH and IGF‐I concentrations, the presented data do not support a significant effect. GH concentrations were statistically different only between months 1 and 12 (single female and twin female) and months 1 and 7 (twin female), showing a decreasing trend. However, GH concentrations remained statistically similar during the other months, making it impossible to conclusively determine an age‐related decline. Similarly, IGF‐I concentrations showed statistical differences only between months 1 and 12 in single males. Therefore, no definitive conclusions can be drawn regarding age‐related increases or decreases for either parameter. Despite the absence of significant age‐related changes in GH and IGF‐I concentrations, correlation analysis revealed a negative relationship between these parameters. This intriguing finding warrants further research to elucidate the underlying mechanisms.

## 5 CONCLUSION

In conclusion, this study is the first to investigate the relationships between growth performance‐related parameters, radiographic measurements and hormonal profiles in Gurcu goats. The physiological, biochemical and growth characteristics of Gurcu goats, a local breed raised in cold climates, have not been fully understood. This study provides important information on age‐related growth performance and radiographic findings in Gurcu goats, contributing to the development of age‐determination methods and the advancement of research in this area. Additionally, age‐related growth performance and hormonal profile differences have not been adequately studied in many goat breeds. These findings could serve as a valuable resource for future research. This context also calls for more comprehensive studies focusing on both environmental and genetic factors in Gurcu goats to better understand the effects of these factors on growth performance and hormonal profiles.

## AUTHOR CONTRIBUTIONS

**Buket Boğa Kuru**: Writing—review and editing; writing—original draft; visualization; validation; supervision; software; resources; project administration; methodology; investigation; formal analysis; data curation; conceptualization. **Enes Akyüz, Uğur Aydın, Mushap Kuru**: Writing—review and editing; visualization; supervision; resources; methodology; data curation; conceptualization. **Fikret Bektaşoğlu, Mert Sezer, Uğur Yıldız**: Writing—review and editing; project administration; methodology; investigation; formal analysis; data curation. **Turgut Kırmızıbayrak**: Writing—review and editing; supervision; methodology. All authors edited, read and approved the final manuscript.

## CONFLICT OF INTEREST STATEMENT

The authors declare no conflicts of interest.

## FUNDING INFORMATION

The authors declare that no funding, grant or other support was received during the conduct of this study and the preparation of this manuscript.

### ETHICS STATEMENT

The Kafkas University Local Ethics Committee for Animal Experiments (KAÜ‐HADYEK/2022‐052), Kars, Türkiye, gave its clearance before this study could be conducted.

### PEER REVIEW

The peer review history for this article is available at https://publons.com/publon/10.1002/vms3.70013.

## Supporting information

Supporting Information

## Data Availability

The data that support the findings of this study are available from the corresponding author upon reasonable request.
