# Supplementary material for: Effect of birth type and sex on growth performance, wither height, humerus‐radius bone dimensions, humerus–ulna growth plate width and selected hormone profile in growing Gurcu goat kids
Source: Vet Med Sci. 2024 Sep 10;10(5):e70013. doi: 10.1002/vms3.70013 (PMC11386322; doi:10.1002/vms3.70013)
Supplement: Supplementary file 1 — Supporting Information [file VMS3-10-e70013-s001.docx]

**Supplementary Tables**

**Supplementary Table 1:** Pairwise comparison of growth performance between groups (single female, single male, twin female, twin male) and within groups across months in Gurcu goat kids.

| **Tukey's multiple comparisons test** | **Mean Diff.** | **95.00% CI of diff.** | **Adjusted P Value** |
| --- | --- | --- | --- |
|  |  |  |  |
| Month 5:Single female vs. Month 5:Single male | -6.070 | -11.38 to -0.7648 | 0.01 |
| Month 5:Single male vs. Month 5:Twin female | 9.640 | 4.335 to 14.95 | <0.001 |
| Month 5:Single male vs. Month 5:Twin male | 8.530 | 3.225 to 13.84 | <0.001 |
| Month 7:Single male vs. Month 7:Twin female | 6.200 | 0.8948 to 11.51 | 0.007 |
| Month 12:Single female vs. Month 12:Single male | -8.400 | -13.71 to -3.095 | <0.001 |
| Month 12:Single male vs. Month 12:Twin female | 7.920 | 2.615 to 13.23 | <0.001 |
|  |  |  |  |
| Month 1:Single female vs. Month 3:Single female | -7.908 | -13.21 to -2.603 | <0.001 |
| Month 1:Single female vs. Month 5:Single female | -14.63 | -19.93 to -9.323 | <0.001 |
| Month 1:Single female vs. Month 7:Single female | -20.18 | -25.48 to -14.87 | <0.001 |
| Month 1:Single female vs. Month 9:Single female | -24.10 | -29.40 to -18.79 | <0.001 |
| Month 1:Single female vs. Month 12:Single female | -26.12 | -31.42 to -20.81 | <0.001 |
| Month 1:Single male vs. Month 3:Single male | -9.068 | -14.37 to -3.763 | <0.001 |
| Month 1:Single male vs. Month 5:Single male | -19.63 | -24.93 to -14.32 | <0.001 |
| Month 1:Single male vs. Month 7:Single male | -25.31 | -30.61 to -20.00 | <0.001 |
| Month 1:Single male vs. Month 9:Single male | -28.15 | -33.45 to -22.84 | <0.001 |
| Month 1:Single male vs. Month 12:Single male | -33.45 | -38.75 to -28.14 | <0.001 |
| Month 1:Twin female vs. Month 5:Twin female | -10.31 | -15.61 to -5.003 | <0.001 |
| Month 1:Twin female vs. Month 7:Twin female | -19.43 | -24.73 to -14.12 | <0.001 |
| Month 1:Twin female vs. Month 9:Twin female | -23.85 | -29.15 to -18.54 | <0.001 |
| Month 1:Twin female vs. Month 12:Twin female | -25.85 | -31.15 to -20.54 | <0.001 |
| Month 1:Twin male vs. Month 5:Twin male | -11.29 | -16.60 to -5.985 | <0.001 |
| Month 1:Twin male vs. Month 7:Twin male | -20.90 | -26.21 to -15.59 | <0.001 |
| Month 1:Twin male vs. Month 9:Twin male | -24.00 | -29.31 to -18.69 | <0.001 |
| Month 1:Twin male vs. Month 12:Twin male | -29.22 | -34.53 to -23.91 | <0.001 |
| Month 3:Single female vs. Month 5:Single female | -6.720 | -12.03 to -1.415 | 0.002 |
| Month 3:Single female vs. Month 7:Single female | -12.27 | -17.58 to -6.965 | <0.001 |
| Month 3:Single female vs. Month 9:Single female | -16.19 | -21.50 to -10.88 | <0.001 |
| Month 3:Single female vs. Month 12:Single female | -18.21 | -23.52 to -12.90 | <0.001 |
| Month 3:Single male vs. Month 5:Single male | -10.56 | -15.87 to -5.255 | <0.001 |
| Month 3:Single male vs. Month 7:Single male | -16.24 | -21.55 to -10.93 | <0.001 |
| Month 3:Single male vs. Month 9:Single male | -19.08 | -24.39 to -13.77 | <0.001 |
| Month 3:Single male vs. Month 12:Single male | -24.38 | -29.69 to -19.07 | <0.001 |
| Month 3:Twin female vs. Month 5:Twin female | -5.866 | -11.17 to -0.5608 | 0.02 |
| Month 3:Twin female vs. Month 7:Twin female | -14.99 | -20.29 to -9.681 | <0.001 |
| Month 3:Twin female vs. Month 9:Twin female | -19.41 | -24.71 to -14.10 | <0.001 |
| Month 3:Twin female vs. Month 12:Twin female | -21.41 | -26.71 to -16.10 | <0.001 |
| Month 3:Twin male vs. Month 5:Twin male | -7.260 | -12.57 to -1.955 | <0.001 |
| Month 3:Twin male vs. Month 7:Twin male | -16.87 | -22.18 to -11.56 | <0.001 |
| Month 3:Twin male vs. Month 9:Twin male | -19.97 | -25.28 to -14.66 | <0.001 |
| Month 3:Twin male vs. Month 12:Twin male | -25.19 | -30.50 to -19.88 | <0.001 |
| Month 5:Single female vs. Month 7:Single female | -5.550 | -10.86 to -0.2448 | 0.03 |
| Month 5:Single female vs. Month 9:Single female | -9.470 | -14.78 to -4.165 | <0.001 |
| Month 5:Single female vs. Month 12:Single female | -11.49 | -16.80 to -6.185 | <0.001 |
| Month 5:Single male vs. Month 7:Single male | -5.680 | -10.99 to -0.3748 | 0.02 |
| Month 5:Single male vs. Month 9:Single male | -8.520 | -13.83 to -3.215 | <0.001 |
| Month 5:Single male vs. Month 12:Single male | -13.82 | -19.13 to -8.515 | <0.001 |
| Month 5:Twin female vs. Month 7:Twin female | -9.120 | -14.43 to -3.815 | <0.001 |
| Month 5:Twin female vs. Month 9:Twin female | -13.54 | -18.85 to -8.235 | <0.001 |
| Month 5:Twin female vs. Month 12:Twin female | -15.54 | -20.85 to -10.23 | <0.001 |
| Month 5:Twin male vs. Month 7:Twin male | -9.610 | -14.92 to -4.305 | <0.001 |
| Month 5:Twin male vs. Month 9:Twin male | -12.71 | -18.02 to -7.405 | <0.001 |
| Month 5:Twin male vs. Month 12:Twin male | -17.93 | -23.24 to -12.62 | <0.001 |
| Month 7:Single female vs. Month 12:Single female | -5.940 | -11.25 to -0.6348 | 0.01 |
| Month 7:Single male vs. Month 12:Single male | -8.140 | -13.45 to -2.835 | <0.001 |
| Month 7:Twin female vs. Month 12:Twin female | -6.420 | -11.73 to -1.115 | 0.004 |
| Month 7:Twin male vs. Month 12:Twin male | -8.320 | -13.63 to -3.015 | <0.001 |
| Month 9:Single male vs. Month 12:Single male | -5.300 | -10.61 to 0.005152 | 0.05 |

Note: Months exhibiting statistically significant differences in pairwise comparisons are presented above. Comparisons without statistically significant differences are not included in the table.

**Supplementary Table 2:** Pairwise comparison of wither height between groups (single female, single male, twin female, twin male) and within groups across months in Gurcu goat kids.

| **Tukey's multiple comparisons test** | **Mean Diff.** | **95.00% CI of diff.** | **Adjusted P Value** |
| --- | --- | --- | --- |
|  |  |  |  |
| Month 3:Single male vs. Month 3:Twin female | 7.100 | 1.895 to 12.31 | <0.001 |
| Month 3:Single male vs. Month 3:Twin male | 6.200 | 0.9946 to 11.41 | 0.006 |
| Month 5:Single female vs. Month 5:Single male | -7.100 | -12.31 to -1.895 | <0.001 |
| Month 5:Single male vs. Month 5:Twin female | 7.800 | 2.595 to 13.01 | <0.001 |
| Month 5:Single male vs. Month 5:Twin male | 6.500 | 1.295 to 11.71 | 0.003 |
| Month 7:Single female vs. Month 7:Single male | -7.000 | -12.21 to -1.795 | <0.001 |
| Month 7:Single male vs. Month 7:Twin female | 5.700 | 0.4946 to 10.91 | 0.02 |
| Month 9:Single female vs. Month 9:Single male | -8.100 | -13.31 to -2.895 | <0.001 |
| Month 9:Single male vs. Month 9:Twin female | 6.700 | 1.495 to 11.91 | 0.002 |
| Month 12:Single female vs. Month 12:Single male | -6.900 | -12.11 to -1.695 | <0.001 |
| Month 12:Single male vs. Month 12:Twin female | 5.800 | 0.5946 to 11.01 | 0.01 |
|  |  |  |  |
| Month 1:Single female vs. Month 3:Single female | -11.40 | -16.61 to -6.195 | <0.001 |
| Month 1:Single female vs. Month 5:Single female | -15.00 | -20.21 to -9.795 | <0.001 |
| Month 1:Single female vs. Month 7:Single female | -17.70 | -22.91 to -12.49 | <0.001 |
| Month 1:Single female vs. Month 9:Single female | -21.00 | -26.21 to -15.79 | <0.001 |
| Month 1:Single female vs. Month 12:Single female | -23.50 | -28.71 to -18.29 | <0.001 |
| Month 1:Single male vs. Month 3:Single male | -11.90 | -17.11 to -6.695 | <0.001 |
| Month 1:Single male vs. Month 5:Single male | -17.70 | -22.91 to -12.49 | <0.001 |
| Month 1:Single male vs. Month 7:Single male | -20.30 | -25.51 to -15.09 | <0.001 |
| Month 1:Single male vs. Month 9:Single male | -24.70 | -29.91 to -19.49 | <0.001 |
| Month 1:Single male vs. Month 12:Single male | -26.00 | -31.21 to -20.79 | <0.001 |
| Month 1:Twin female vs. Month 3:Twin female | -6.100 | -11.31 to -0.8946 | 0.007 |
| Month 1:Twin female vs. Month 5:Twin female | -11.20 | -16.41 to -5.995 | <0.001 |
| Month 1:Twin female vs. Month 7:Twin female | -15.90 | -21.11 to -10.69 | <0.001 |
| Month 1:Twin female vs. Month 9:Twin female | -19.30 | -24.51 to -14.09 | <0.001 |
| Month 1:Twin female vs. Month 12:Twin female | -21.50 | -26.71 to -16.29 | <0.001 |
| Month 1:Twin male vs. Month 3:Twin male | -8.600 | -13.81 to -3.395 | <0.001 |
| Month 1:Twin male vs. Month 5:Twin male | -14.10 | -19.31 to -8.895 | <0.001 |
| Month 1:Twin male vs. Month 7:Twin male | -19.50 | -24.71 to -14.29 | <0.001 |
| Month 1:Twin male vs. Month 9:Twin male | -23.10 | -28.31 to -17.89 | <0.001 |
| Month 1:Twin male vs. Month 12:Twin male | -26.60 | -31.81 to -21.39 | <0.001 |
| Month 3:Single female vs. Month 7:Single female | -6.300 | -11.51 to -1.095 | 0.004 |
| Month 3:Single female vs. Month 9:Single female | -9.600 | -14.81 to -4.395 | <0.001 |
| Month 3:Single female vs. Month 12:Single female | -12.10 | -17.31 to -6.895 | <0.001 |
| Month 3:Single male vs. Month 5:Single male | -5.800 | -11.01 to -0.5946 | 0.01 |
| Month 3:Single male vs. Month 7:Single male | -8.400 | -13.61 to -3.195 | <0.001 |
| Month 3:Single male vs. Month 9:Single male | -12.80 | -18.01 to -7.595 | <0.001 |
| Month 3:Single male vs. Month 12:Single male | -14.10 | -19.31 to -8.895 | <0.001 |
| Month 3:Twin female vs. Month 7:Twin female | -9.800 | -15.01 to -4.595 | <0.001 |
| Month 3:Twin female vs. Month 9:Twin female | -13.20 | -18.41 to -7.995 | <0.001 |
| Month 3:Twin female vs. Month 12:Twin female | -15.40 | -20.61 to -10.19 | <0.001 |
| Month 3:Twin male vs. Month 5:Twin male | -5.500 | -10.71 to -0.2946 | 0.03 |
| Month 3:Twin male vs. Month 7:Twin male | -10.90 | -16.11 to -5.695 | <0.001 |
| Month 3:Twin male vs. Month 9:Twin male | -14.50 | -19.71 to -9.295 | <0.001 |
| Month 3:Twin male vs. Month 12:Twin male | -18.00 | -23.21 to -12.79 | <0.001 |
| Month 5:Single female vs. Month 9:Single female | -6.000 | -11.21 to -0.7946 | 0.009 |
| Month 5:Single female vs. Month 12:Single female | -8.500 | -13.71 to -3.295 | <0.001 |
| Month 5:Single male vs. Month 9:Single male | -7.000 | -12.21 to -1.795 | <0.001 |
| Month 5:Single male vs. Month 12:Single male | -8.300 | -13.51 to -3.095 | <0.001 |
| Month 5:Twin female vs. Month 9:Twin female | -8.100 | -13.31 to -2.895 | <0.001 |
| Month 5:Twin female vs. Month 12:Twin female | -10.30 | -15.51 to -5.095 | <0.001 |
| Month 5:Twin male vs. Month 7:Twin male | -5.400 | -10.61 to -0.1946 | 0.03 |
| Month 5:Twin male vs. Month 9:Twin male | -9.000 | -14.21 to -3.795 | <0.001 |
| Month 5:Twin male vs. Month 12:Twin male | -12.50 | -17.71 to -7.295 | <0.001 |
| Month 7:Single female vs. Month 12:Single female | -5.800 | -11.01 to -0.5946 | 0.01 |
| Month 7:Single male vs. Month 12:Single male | -5.700 | -10.91 to -0.4946 | 0.02 |
| Month 7:Twin female vs. Month 12:Twin female | -5.600 | -10.81 to -0.3946 | 0.02 |
| Month 7:Twin male vs. Month 12:Twin male | -7.100 | -12.31 to -1.895 | <0.001 |

Note: Months exhibiting statistically significant differences in pairwise comparisons are presented above. Comparisons without statistically significant differences are not included in the table.

**Supplementary Table 3:** Pairwise comparison of humerus length between groups (single female, single male, twin female, twin male) and within groups over months in Gurcu goat kids.

| **Tukey's multiple comparisons test** | **Mean Diff.** | **95.00% CI of diff.** | **Adjusted P Value** |
| --- | --- | --- | --- |
|  |  |  |  |
| Month 7:Single female vs. Month 7:Single male | -1.204 | -2.336 to -0.07153 | 0.03 |
| Month 9:Single female vs. Month 9:Single male | -1.150 | -2.282 to -0.01753 | 0.05 |
|  |  |  |  |
| Month 1:Single female vs. Month 3:Single female | -2.004 | -3.403 to -0.6048 | <0.001 |
| Month 1:Single female vs. Month 5:Single female | -3.626 | -5.025 to -2.227 | <0.001 |
| Month 1:Single female vs. Month 7:Single female | -4.190 | -5.589 to -2.791 | <0.001 |
| Month 1:Single female vs. Month 9:Single female | -4.630 | -6.029 to -3.231 | <0.001 |
| Month 1:Single female vs. Month 12:Single female | -5.868 | -7.267 to -4.469 | <0.001 |
| Month 1:Single male vs. Month 3:Single male | -2.816 | -4.215 to -1.417 | <0.001 |
| Month 1:Single male vs. Month 5:Single male | -4.378 | -5.777 to -2.979 | <0.001 |
| Month 1:Single male vs. Month 7:Single male | -5.684 | -7.083 to -4.285 | <0.001 |
| Month 1:Single male vs. Month 9:Single male | -6.070 | -7.469 to -4.671 | <0.001 |
| Month 1:Single male vs. Month 12:Single male | -7.192 | -8.591 to -5.793 | <0.001 |
| Month 1:Twin female vs. Month 3:Twin female | -1.806 | -3.205 to -0.4068 | 0.002 |
| Month 1:Twin female vs. Month 5:Twin female | -3.794 | -5.193 to -2.395 | <0.001 |
| Month 1:Twin female vs. Month 7:Twin female | -4.490 | -5.889 to -3.091 | <0.001 |
| Month 1:Twin female vs. Month 9:Twin female | -5.070 | -6.469 to -3.671 | <0.001 |
| Month 1:Twin female vs. Month 12:Twin female | -6.260 | -7.659 to -4.861 | <0.001 |
| Month 1:Twin male vs. Month 3:Twin male | -1.920 | -3.319 to -0.5208 | <0.001 |
| Month 1:Twin male vs. Month 5:Twin male | -3.622 | -5.021 to -2.223 | <0.001 |
| Month 1:Twin male vs. Month 7:Twin male | -5.156 | -6.555 to -3.757 | <0.001 |
| Month 1:Twin male vs. Month 9:Twin male | -5.444 | -6.843 to -4.045 | <0.001 |
| Month 1:Twin male vs. Month 12:Twin male | -6.718 | -8.117 to -5.319 | <0.001 |
| Month 3:Single female vs. Month 5:Single female | -1.622 | -3.021 to -0.2228 | 0.008 |
| Month 3:Single female vs. Month 7:Single female | -2.186 | -3.585 to -0.7868 | <0.001 |
| Month 3:Single female vs. Month 9:Single female | -2.626 | -4.025 to -1.227 | <0.001 |
| Month 3:Single female vs. Month 12:Single female | -3.864 | -5.263 to -2.465 | <0.001 |
| Month 3:Single male vs. Month 5:Single male | -1.562 | -2.961 to -0.1628 | 0.01 |
| Month 3:Single male vs. Month 7:Single male | -2.868 | -4.267 to -1.469 | <0.001 |
| Month 3:Single male vs. Month 9:Single male | -3.254 | -4.653 to -1.855 | <0.001 |
| Month 3:Single male vs. Month 12:Single male | -4.376 | -5.775 to -2.977 | <0.001 |
| Month 3:Twin female vs. Month 5:Twin female | -1.988 | -3.387 to -0.5888 | <0.001 |
| Month 3:Twin female vs. Month 7:Twin female | -2.684 | -4.083 to -1.285 | <0.001 |
| Month 3:Twin female vs. Month 9:Twin female | -3.264 | -4.663 to -1.865 | <0.001 |
| Month 3:Twin female vs. Month 12:Twin female | -4.454 | -5.853 to -3.055 | <0.001 |
| Month 3:Twin male vs. Month 5:Twin male | -1.702 | -3.101 to -0.3028 | 0.004 |
| Month 3:Twin male vs. Month 7:Twin male | -3.236 | -4.635 to -1.837 | <0.001 |
| Month 3:Twin male vs. Month 9:Twin male | -3.524 | -4.923 to -2.125 | <0.001 |
| Month 3:Twin male vs. Month 12:Twin male | -4.798 | -6.197 to -3.399 | <0.001 |
| Month 5:Single female vs. Month 12:Single female | -2.242 | -3.641 to -0.8428 | <0.001 |
| Month 5:Single male vs. Month 9:Single male | -1.692 | -3.091 to -0.2928 | 0.004 |
| Month 5:Single male vs. Month 12:Single male | -2.814 | -4.213 to -1.415 | <0.001 |
| Month 5:Twin female vs. Month 12:Twin female | -2.466 | -3.865 to -1.067 | <0.001 |
| Month 5:Twin male vs. Month 7:Twin male | -1.534 | -2.933 to -0.1348 | 0.02 |
| Month 5:Twin male vs. Month 9:Twin male | -1.822 | -3.221 to -0.4228 | 0.001 |
| Month 5:Twin male vs. Month 12:Twin male | -3.096 | -4.495 to -1.697 | <0.001 |
| Month 7:Single female vs. Month 12:Single female | -1.678 | -3.077 to -0.2788 | 0.005 |
| Month 7:Single male vs. Month 12:Single male | -1.508 | -2.907 to -0.1088 | 0.02 |
| Month 7:Twin female vs. Month 12:Twin female | -1.770 | -3.169 to -0.3708 | 0.002 |
| Month 7:Twin male vs. Month 12:Twin male | -1.562 | -2.961 to -0.1628 | 0.01 |

Note: Months exhibiting statistically significant differences in pairwise comparisons are presented above. Comparisons without statistically significant differences are not included in the table.

**Supplementary Table 4:** Pairwise comparison of radius length within groups over months in Gurcu goat kids

| **Tukey's multiple comparisons test** | **Mean Diff.** | **95.00% CI of diff.** | **Adjusted P Value** |
| --- | --- | --- | --- |
| Month 1:Single female vs. Month 5:Single female | -2.816 | -4.386 to -1.246 | <0.001 |
| Month 1:Single female vs. Month 7:Single female | -4.236 | -5.806 to -2.666 | <0.001 |
| Month 1:Single female vs. Month 9:Single female | -4.520 | -6.090 to -2.950 | <0.001 |
| Month 1:Single female vs. Month 12:Single female | -5.418 | -6.988 to -3.848 | <0.001 |
| Month 1:Single male vs. Month 3:Single male | -1.902 | -3.472 to -0.3324 | 0.004 |
| Month 1:Single male vs. Month 5:Single male | -3.460 | -5.030 to -1.890 | <0.001 |
| Month 1:Single male vs. Month 7:Single male | -4.946 | -6.516 to -3.376 | <0.001 |
| Month 1:Single male vs. Month 9:Single male | -5.310 | -6.880 to -3.740 | <0.001 |
| Month 1:Single male vs. Month 12:Single male | -5.914 | -7.484 to -4.344 | <0.001 |
| Month 1:Twin female vs. Month 5:Twin female | -3.158 | -4.728 to -1.588 | <0.001 |
| Month 1:Twin female vs. Month 7:Twin female | -4.436 | -6.006 to -2.866 | <0.001 |
| Month 1:Twin female vs. Month 9:Twin female | -4.830 | -6.400 to -3.260 | <0.001 |
| Month 1:Twin female vs. Month 12:Twin female | -5.596 | -7.166 to -4.026 | <0.001 |
| Month 1:Twin male vs. Month 5:Twin male | -3.214 | -4.784 to -1.644 | <0.001 |
| Month 1:Twin male vs. Month 7:Twin male | -4.458 | -6.028 to -2.888 | <0.001 |
| Month 1:Twin male vs. Month 9:Twin male | -4.878 | -6.448 to -3.308 | <0.001 |
| Month 1:Twin male vs. Month 12:Twin male | -6.152 | -7.722 to -4.582 | <0.001 |
| Month 3:Single female vs. Month 7:Single female | -2.960 | -4.530 to -1.390 | <0.001 |
| Month 3:Single female vs. Month 9:Single female | -3.244 | -4.814 to -1.674 | <0.001 |
| Month 3:Single female vs. Month 12:Single female | -4.142 | -5.712 to -2.572 | <0.001 |
| Month 3:Single male vs. Month 5:Single male | -1.558 | -3.128 to 0.01158 | 0.05 |
| Month 3:Single male vs. Month 7:Single male | -3.044 | -4.614 to -1.474 | <0.001 |
| Month 3:Single male vs. Month 9:Single male | -3.408 | -4.978 to -1.838 | <0.001 |
| Month 3:Single male vs. Month 12:Single male | -4.012 | -5.582 to -2.442 | <0.001 |
| Month 3:Twin female vs. Month 5:Twin female | -2.198 | -3.768 to -0.6284 | <0.001 |
| Month 3:Twin female vs. Month 7:Twin female | -3.476 | -5.046 to -1.906 | <0.001 |
| Month 3:Twin female vs. Month 9:Twin female | -3.870 | -5.440 to -2.300 | <0.001 |
| Month 3:Twin female vs. Month 12:Twin female | -4.636 | -6.206 to -3.066 | <0.001 |
| Month 3:Twin male vs. Month 5:Twin male | -2.176 | -3.746 to -0.6064 | <0.001 |
| Month 3:Twin male vs. Month 7:Twin male | -3.420 | -4.990 to -1.850 | <0.001 |
| Month 3:Twin male vs. Month 9:Twin male | -3.840 | -5.410 to -2.270 | <0.001 |
| Month 3:Twin male vs. Month 12:Twin male | -5.114 | -6.684 to -3.544 | <0.001 |
| Month 5:Single female vs. Month 9:Single female | -1.704 | -3.274 to -0.1344 | 0.02 |
| Month 5:Single female vs. Month 12:Single female | -2.602 | -4.172 to -1.032 | <0.001 |
| Month 5:Single male vs. Month 9:Single male | -1.850 | -3.420 to -0.2804 | 0.006 |
| Month 5:Single male vs. Month 12:Single male | -2.454 | -4.024 to -0.8844 | <0.001 |
| Month 5:Twin female vs. Month 9:Twin female | -1.672 | -3.242 to -0.1024 | 0.02 |
| Month 5:Twin female vs. Month 12:Twin female | -2.438 | -4.008 to -0.8684 | <0.001 |
| Month 5:Twin male vs. Month 9:Twin male | -1.664 | -3.234 to -0.09442 | 0.03 |
| Month 5:Twin male vs. Month 12:Twin male | -2.938 | -4.508 to -1.368 | <0.001 |
| Month 7:Twin male vs. Month 12:Twin male | -1.694 | -3.264 to -0.1244 | 0.02 |

Note: Months exhibiting statistically significant differences in pairwise comparisons are presented above. Comparisons without statistically significant differences are not included in the table.

**Supplementary Table 5:** Inter-group (single female, single male, twin female, twin male) and intra-group monthly comparison of humeral epiphyseal plate width in Gurcu goat kids.

| **Tukey's multiple comparisons test** | **Mean Diff.** | **95.00% CI of diff.** | **Adjusted P Value** |
| --- | --- | --- | --- |
|  |  |  |  |
| Month 1:Single female vs. Month 1:Twin female | -0.4200 | -0.7462 to -0.09378 | 0.006 |
|  |  |  |  |
| Month 1:Single female vs. Month 3:Single female | 1.140 | 0.7484 to 1.532 | <0.001 |
| Month 1:Single female vs. Month 5:Single female | 1.280 | 0.8884 to 1.672 | <0.001 |
| Month 1:Single female vs. Month 7:Single female | 1.420 | 1.028 to 1.812 | <0.001 |
| Month 1:Single female vs. Month 9:Single female | 1.480 | 1.088 to 1.872 | <0.001 |
| Month 1:Single female vs. Month 12:Single female | 1.900 | 1.508 to 2.292 | <0.001 |
| Month 1:Single male vs. Month 3:Single male | 1.220 | 0.8284 to 1.612 | <0.001 |
| Month 1:Single male vs. Month 5:Single male | 1.460 | 1.068 to 1.852 | <0.001 |
| Month 1:Single male vs. Month 7:Single male | 1.620 | 1.228 to 2.012 | <0.001 |
| Month 1:Single male vs. Month 9:Single male | 1.740 | 1.348 to 2.132 | <0.001 |
| Month 1:Single male vs. Month 12:Single male | 1.960 | 1.568 to 2.352 | <0.001 |
| Month 1:Twin female vs. Month 3:Twin female | 1.460 | 1.068 to 1.852 | <0.001 |
| Month 1:Twin female vs. Month 5:Twin female | 1.660 | 1.268 to 2.052 | <0.001 |
| Month 1:Twin female vs. Month 7:Twin female | 1.800 | 1.408 to 2.192 | <0.001 |
| Month 1:Twin female vs. Month 9:Twin female | 1.940 | 1.548 to 2.332 | <0.001 |
| Month 1:Twin female vs. Month 12:Twin female | 2.220 | 1.828 to 2.612 | <0.001 |
| Month 1:Twin male vs. Month 3:Twin male | 1.220 | 0.8284 to 1.612 | <0.001 |
| Month 1:Twin male vs. Month 5:Twin male | 1.360 | 0.9684 to 1.752 | <0.001 |
| Month 1:Twin male vs. Month 7:Twin male | 1.440 | 1.048 to 1.832 | <0.001 |
| Month 1:Twin male vs. Month 9:Twin male | 1.600 | 1.208 to 1.992 | <0.001 |
| Month 1:Twin male vs. Month 12:Twin male | 1.980 | 1.588 to 2.372 | <0.001 |
| Month 3:Single female vs. Month 12:Single female | 0.7600 | 0.3684 to 1.152 | <0.001 |
| Month 3:Single male vs. Month 7:Single male | 0.4000 | 0.008445 to 0.7916 | 0.04 |
| Month 3:Single male vs. Month 9:Single male | 0.5200 | 0.1284 to 0.9116 | <0.001 |
| Month 3:Single male vs. Month 12:Single male | 0.7400 | 0.3484 to 1.132 | <0.001 |
| Month 3:Twin female vs. Month 9:Twin female | 0.4800 | 0.08844 to 0.8716 | 0.004 |
| Month 3:Twin female vs. Month 12:Twin female | 0.7600 | 0.3684 to 1.152 | <0.001 |
| Month 3:Twin male vs. Month 12:Twin male | 0.7600 | 0.3684 to 1.152 | <0.001 |
| Month 5:Single female vs. Month 12:Single female | 0.6200 | 0.2284 to 1.012 | <0.001 |
| Month 5:Single male vs. Month 12:Single male | 0.5000 | 0.1084 to 0.8916 | 0.002 |
| Month 5:Twin female vs. Month 12:Twin female | 0.5600 | 0.1684 to 0.9516 | <0.001 |
| Month 5:Twin male vs. Month 12:Twin male | 0.6200 | 0.2284 to 1.012 | <0.001 |
| Month 7:Single female vs. Month 12:Single female | 0.4800 | 0.08844 to 0.8716 | 0.004 |
| Month 7:Twin female vs. Month 12:Twin female | 0.4200 | 0.02844 to 0.8116 | 0.02 |
| Month 7:Twin male vs. Month 12:Single male | 0.4000 | 0.008445 to 0.7916 | 0.04 |
| Month 7:Twin male vs. Month 12:Twin male | 0.5400 | 0.1484 to 0.9316 | <0.001 |
| Month 9:Single female vs. Month 12:Single female | 0.4200 | 0.02844 to 0.8116 | 0.02 |

Note: Months exhibiting statistically significant differences in pairwise comparisons are presented above. Comparisons without statistically significant differences are not included in the table.

**Supplementary Table 6:** Intra-group monthly comparison of ulnar epiphyseal plate width in Gurcu goat kids.

| **Tukey's multiple comparisons test** | **Mean Diff.** | **95.00% CI of diff.** | **Adjusted P Value** |
| --- | --- | --- | --- |
| Month 1:Single female vs. Month 3:Single female | 1.060 | 0.6232 to 1.497 | <0.001 |
| Month 1:Single female vs. Month 5:Single female | 1.820 | 1.383 to 2.257 | <0.001 |
| Month 1:Single female vs. Month 7:Single female | 2.040 | 1.603 to 2.477 | <0.001 |
| Month 1:Single female vs. Month 9:Single female | 2.220 | 1.783 to 2.657 | <0.001 |
| Month 1:Single female vs. Month 12:Single female | 2.460 | 2.023 to 2.897 | <0.001 |
| Month 1:Single male vs. Month 3:Single male | 1.180 | 0.7432 to 1.617 | <0.001 |
| Month 1:Single male vs. Month 5:Single male | 2.120 | 1.683 to 2.557 | <0.001 |
| Month 1:Single male vs. Month 7:Single male | 2.320 | 1.883 to 2.757 | <0.001 |
| Month 1:Single male vs. Month 9:Single male | 2.540 | 2.103 to 2.977 | <0.001 |
| Month 1:Single male vs. Month 12:Single male | 2.800 | 2.363 to 3.237 | <0.001 |
| Month 1:Twin female vs. Month 3:Twin female | 1.080 | 0.6432 to 1.517 | <0.001 |
| Month 1:Twin female vs. Month 5:Twin female | 1.820 | 1.383 to 2.257 | <0.001 |
| Month 1:Twin female vs. Month 7:Twin female | 2.100 | 1.663 to 2.537 | <0.001 |
| Month 1:Twin female vs. Month 9:Twin female | 2.260 | 1.823 to 2.697 | <0.001 |
| Month 1:Twin female vs. Month 12:Twin female | 2.400 | 1.963 to 2.837 | <0.001 |
| Month 1:Twin male vs. Month 3:Twin male | 1.120 | 0.6832 to 1.557 | <0.001 |
| Month 1:Twin male vs. Month 5:Twin male | 1.760 | 1.323 to 2.197 | <0.001 |
| Month 1:Twin male vs. Month 7:Twin male | 1.960 | 1.523 to 2.397 | <0.001 |
| Month 1:Twin male vs. Month 9:Twin male | 2.160 | 1.723 to 2.597 | <0.001 |
| Month 1:Twin male vs. Month 12:Twin male | 2.420 | 1.983 to 2.857 | <0.001 |
| Month 3:Single female vs. Month 5:Single female | 0.7600 | 0.3232 to 1.197 | <0.001 |
| Month 3:Single female vs. Month 7:Single female | 0.9800 | 0.5432 to 1.417 | <0.001 |
| Month 3:Single female vs. Month 9:Single female | 1.160 | 0.7232 to 1.597 | <0.001 |
| Month 3:Single female vs. Month 12:Single female | 1.400 | 0.9632 to 1.837 | <0.001 |
| Month 3:Single male vs. Month 5:Single male | 0.9400 | 0.5032 to 1.377 | <0.001 |
| Month 3:Single male vs. Month 7:Single male | 1.140 | 0.7032 to 1.577 | <0.001 |
| Month 3:Single male vs. Month 9:Single male | 1.360 | 0.9232 to 1.797 | <0.001 |
| Month 3:Single male vs. Month 12:Single male | 1.620 | 1.183 to 2.057 | <0.001 |
| Month 3:Twin female vs. Month 5:Twin female | 0.7400 | 0.3032 to 1.177 | <0.001 |
| Month 3:Twin female vs. Month 7:Twin female | 1.020 | 0.5832 to 1.457 | <0.001 |
| Month 3:Twin female vs. Month 9:Twin female | 1.180 | 0.7432 to 1.617 | <0.001 |
| Month 3:Twin female vs. Month 12:Twin female | 1.320 | 0.8832 to 1.757 | <0.001 |
| Month 3:Twin male vs. Month 5:Twin male | 0.6400 | 0.2032 to 1.077 | <0.001 |
| Month 3:Twin male vs. Month 7:Twin male | 0.8400 | 0.4032 to 1.277 | <0.001 |
| Month 3:Twin male vs. Month 9:Twin male | 1.040 | 0.6032 to 1.477 | <0.001 |
| Month 3:Twin male vs. Month 12:Twin male | 1.300 | 0.8632 to 1.737 | <0.001 |
| Month 5:Single female vs. Month 12:Single female | 0.6400 | 0.2032 to 1.077 | <0.001 |
| Month 5:Single male vs. Month 12:Single male | 0.6800 | 0.2432 to 1.117 | <0.001 |
| Month 5:Twin female vs. Month 9:Twin female | 0.4400 | 0.003233 to 0.8768 | 0.05 |
| Month 5:Twin female vs. Month 12:Twin female | 0.5800 | 0.1432 to 1.017 | <0.001 |
| Month 5:Twin male vs. Month 12:Twin male | 0.6600 | 0.2232 to 1.097 | <0.001 |
| Month 7:Single male vs. Month 12:Single male | 0.4800 | 0.04323 to 0.9168 | 0.02 |
| Month 7:Twin male vs. Month 12:Twin male | 0.4600 | 0.02323 to 0.8968 | 0.03 |

Note: Months exhibiting statistically significant differences in pairwise comparisons are presented above. Comparisons without statistically significant differences are not included in the table.

**Supplementary Table 7:** A pairwise comparison of calcitonin concentration in Gurcu goat kids between groups (single female, single male, twin female, twin male) and between months within groups.

| **Tukey's multiple comparisons test** | **Mean Diff.** | **95.00% CI of diff.** | **Adjusted P Value** |
| --- | --- | --- | --- |
|  |  |  |  |
| Month 5:Single male vs. Month 5:Twin female | 2.098 | 0.3458 to 3.850 | 0.005 |
| Month 5:Single male vs. Month 5:Twin male | 1.954 | 0.2018 to 3.706 | 0.01 |
|  |  |  |  |
| Month 1:Single female vs. Month 7:Single female | 2.256 | 0.5038 to 4.008 | 0.002 |
| Month 1:Single female vs. Month 9:Single female | 2.610 | 0.8578 to 4.362 | <0.001 |
| Month 1:Single female vs. Month 12:Single female | 2.882 | 1.130 to 4.634 | <0.001 |
| Month 1:Single male vs. Month 12:Single male | 2.846 | 1.094 to 4.598 | <0.001 |
| Month 1:Twin male vs. Month 9:Twin male | 2.162 | 0.4098 to 3.914 | 0.003 |
| Month 1:Twin male vs. Month 12:Twin male | 2.410 | 0.6578 to 4.162 | <0.001 |
| Month 3:Single female vs. Month 9:Single female | 1.812 | 0.05980 to 3.564 | 0.03 |
| Month 3:Single female vs. Month 12:Single female | 2.084 | 0.3318 to 3.836 | 0.006 |
| Month 3:Single male vs. Month 12:Single male | 2.774 | 1.022 to 4.526 | <0.001 |
| Month 5:Single female vs. Month 12:Single female | 1.800 | 0.04780 to 3.552 | 0.04 |
| Month 5:Single male vs. Month 12:Single male | 2.774 | 1.022 to 4.526 | <0.001 |

Note: Months exhibiting statistically significant differences in pairwise comparisons are presented above. Comparisons without statistically significant differences are not included in the table.

**Supplementary Table 8:** Pairwise comparison of free triiodothyronine concentration between intra-group months in Gurcu goat kids.

| **Tukey's multiple comparisons test** | **Mean Diff.** | **95.00% CI of diff.** | **Adjusted P Value** |
| --- | --- | --- | --- |
| Month 1:Single female vs. Month 12:Single female | 3.538 | 0.1768 to 6.899 | 0.03 |
| Month 1:Single male vs. Month 12:Single male | 4.290 | 0.9288 to 7.651 | 0.002 |
| Month 1:Twin female vs. Month 12:Twin female | 4.658 | 1.297 to 8.019 | <0.001 |
| Month 1:Twin male vs. Month 12:Twin male | 4.466 | 1.105 to 7.827 | <0.001 |
| Month 3:Single male vs. Month 12:Single male | 3.952 | 0.5908 to 7.313 | 0.007 |
| Month 3:Twin female vs. Month 12:Twin female | 3.752 | 0.3908 to 7.113 | 0.01 |
| Month 5:Twin female vs. Month 12:Twin female | 3.448 | 0.08685 to 6.809 | 0.04 |

Note: Months exhibiting statistically significant differences in pairwise comparisons are presented above. Comparisons without statistically significant differences are not included in the table.

**Supplementary Table 9:** Pairwise comparison of free triiodothyronine / free thyroxine ratio between intra-group months in Gurcu goat kids.

| **Tukey's multiple comparisons test** | **Mean Diff,** | **95,00% CI of diff,** | **Adjusted P Value** |
| --- | --- | --- | --- |
| Month 1:Twin female vs. Month 12:Twin female | 3.000 | 0.05609 to 5,944 | 0.04 |
| Month 3:Single male vs. Month 12:Single male | 2.844 | -0.09991 to 5,788 | 0.05 |

Note: Months exhibiting statistically significant differences in pairwise comparisons are presented above. Comparisons without statistically significant differences are not included in the table.

**Supplementary Table 10:** Pairwise comparison of growth hormone concentration between intra-group months in Gurcu goat kids.

| **Tukey's multiple comparisons test** | **Mean Diff.** | **95.00% CI of diff.** | **Adjusted P Value** |
| --- | --- | --- | --- |
| Month 1:Single female vs. Month 12:Single female | 0.5440 | 0.001485 to 1.087 | 0.05 |
| Month 1:Twin female vs. Month 7:Twin female | 0.5460 | 0.003485 to 1.089 | 0.05 |
| Month 1:Twin female vs. Month 12:Twin female | 0.5720 | 0.02948 to 1.115 | 0.03 |

Note: Months exhibiting statistically significant differences in pairwise comparisons are presented above. Comparisons without statistically significant differences are not included in the table.

**Supplementary Table 11:** Intra-group pairwise comparison of insulin-like growth factor-I concentration in Gurcu goat kids between months.

| **Tukey's multiple comparisons test** | **Mean Diff.** | **95.00% CI of diff.** | **Adjusted P Value** |
| --- | --- | --- | --- |
| Month 1:Single male vs. Month 12:Single male | -50.24 | -103.6 to 3.102 | 0.05 |

Note: Months exhibiting statistically significant differences in pairwise comparisons are presented above. Comparisons without statistically significant differences are not included in the table.
